# Blood vessels guide Schwann cell migration in the adult demyelinated CNS through Eph/ephrin signaling

**DOI:** 10.1007/s00401-019-02011-1

**Published:** 2019-04-22

**Authors:** Beatriz Garcia-Diaz, Corinne Bachelin, Fanny Coulpier, Gaspard Gerschenfeld, Cyrille Deboux, Violetta Zujovic, Patrick Charnay, Piotr Topilko, Anne Baron-Van Evercooren

**Affiliations:** 1 Institut du Cerveau et de la Moelle Epinière-Groupe Hospitalier Pitié-Salpêtrière, INSERM, U1127; CNRS, UMR 7225; Sorbonne Universités, Université Pierre et Marie Curie Paris 06, UM-75, Paris, France; 2 Unidad de Gestión Clínica de Neurociencias, IBIMA, Hospital Regional Universitario de Málaga, Malaga, 29009 Spain; 3 Ecole normale supérieure, PSL Research University, CNRS, Inserm, Institut de Biologie de l’Ecole normale supérieure (IBENS), 75005 Paris, France

**Keywords:** Schwann cells, Central nervous system, Blood vessels, Migration, EphrinB3

## Abstract

Schwann cells (SC) enter the central nervous system (CNS) in pathophysiological conditions. However, how SC invade the CNS to remyelinate central axons remains undetermined. We studied SC migratory behavior ex vivo and in vivo after exogenous transplantation in the demyelinated spinal cord. The data highlight for the first time that SC migrate preferentially along blood vessels in perivascular extracellular matrix (ECM), avoiding CNS myelin. We demonstrate in vitro and in vivo that this migration route occurs by virtue of a dual mode of action of Eph/ephrin signaling. Indeed, EphrinB3, enriched in myelin, interacts with SC Eph receptors, to drive SC away from CNS myelin, and triggers their preferential adhesion to ECM components, such as fibronectin via integrinβ1 interactions. This complex interplay enhances SC migration along the blood vessel network and together with lesion-induced vascular remodeling facilitates their timely invasion of the lesion site. These novel findings elucidate the mechanism by which SC invade and contribute to spinal cord repair.

**Electronic supplementary material:**

The online version of this article (10.1007/s00401-019-02011-1) contains supplementary material, which is available to authorized users.

## Introduction

Myelination, the evolutionary characteristic acquired by vertebrates to allow rapid and saltatory nerve conduction, is supported by two different glial cell types, oligodendrocytes in the central nervous system (CNS), and Schwann cells (SC) in the peripheral nervous system (PNS). These two cell types are mutually exclusive in physiological conditions. However, in demyelinating diseases or injury, this PNS/CNS segregation is compromised: SC can invade and repair the CNS [24, 30], while oligodendrocytes can myelinate peripheral nerve root axons [18]. Remyelination of CNS axons by SC protects axons, restores axonal conduction and even reverses neurological deficits [26], highlighting their potential to rescue the injured CNS [62]. These remyelinating SC arise either from the PNS [6, 43], or are generated from adult oligodendrocyte precursors cells (OPC) [59, 61]. However, SC remyelination of CNS axons is always restricted to the spinal root’s entry and exit zones (reviewed in [62]), and the presence of peripheral myelin has been frequently observed close to blood vessels (BV) [24]. These observations suggest that although SC migrate efficiently in vitro [11, 45] and in vivo [16] into the PNS, their survival and migration within the CNS are limited. The presence of peripheral myelin close to BV raises the possibility for BV to play a role in guiding SC movements within the CNS. While migration along BV has recently been described for different CNS cell types [15, 28, 58] including for SC along regenerating nerves [16], their role in SC invasion of the CNS has not been explored.

CNS white matter [9, 17, 32] inhibits SC migration. Among the molecules involved in various cell-type segregation and guidance within the CNS, the Eph/ephrin family is implicated in both developmental [41, 57] and pathological conditions [47]. In particular, EphrinB3 is expressed in myelin in brain and mouse spinal cord [12, 23, 55], and plays an important role in preventing neurite outgrowth [12], axonal regeneration [23] and OPC  differentiation [55]. EphrinB3 also acts as a repellent molecule in the guidance of axon tracts in the spinal cord during development [49]. Hence, these receptors could also mediate similar SC repulsion by myelin-associated EphrinB3. In addition, EphB is involved in SC sorting and migration in regenerating peripheral nerves [51]. Eph/ephrin signaling modulates cell–cell adhesion, which results in increased integrin-mediated adhesion of Eph/ephrin-expressing cells [5, 20] or, as in the case of EphB signaling, in cell sorting by re-localization of N-cadherin in SC [51]. Furthermore, EphrinB ligands control cell migration through positive adhesion to substrates such as collagen and fibronectin (FN) [54, 60], main components of perivascular extracellular matrix (ECM). Therefore, these cues could influence the capacity of SC to migrate within the CNS and/or interact with CNS myelin.

In spite of these observations, a role for BV in SC migration/recruitment in the CNS and for EphrinB3 in modulating SC interactions with CNS myelin and/or BV has not been investigated. Using ex vivo, in vivo and in vitro paradigms, we show that BV are a preferred substrate for SC migration into the CNS. Moreover, we establish that CNS myelin plays an essential role in SC exclusion from the CNS and demonstrate that this effect is partially mediated by EphrinB3. Finally, myelin-associated EphrinB3 modulates SC adhesion to the ECM component FN, via interactions with integrinβ1. This increased SC–FN adhesion to perivascular ECM overrules SC inhibition by myelin and promotes SC migration along BV, facilitating their arrival at the lesion. Thus, Eph/ephrin may guide SC within the CNS according to a dual mode of action, repulsing SC from CNS white matter on one hand, and favoring their interaction with BV on the other. These observations shed new light on the mechanism of SC invasion into the damaged CNS.

## Materials and methods

*Animals* 8-week-old C57Bl/6JR female mice were purchased from Janvier Labs (Rodent Research Models and Associated Services) and used for engraftment. SC were obtained from green fluorescent protein-tagged actin (GFP^+^) transgenic mice, *Krox20*^*Cre/*+^*R26R*^*YFP/*+^ mice, and TdTomato *Krox20*^*Cre/flox*^*,R26*^*mT/*+^ mice which were previously characterized [18, 44], and maintained at ICM and IBENS animal facilities. Animal experiments were performed according to European Community regulations, ICM and INSERM ethical committee (authorization 75-348; 20/04/2005) and were approved by the local Darwin ethical committee.

* SC isolation and purification* Sciatic nerves from GFP^+^ mice were isolated at postnatal day 15. Purification procedure was adapted from the previously described protocol [7]. Briefly, enzymatic dissociation was performed by incubation with trypsin 0.025% and collagenase (420 U/ml) for 10 min at 37 °C, followed by mechanical dissociation through different needle gauges. After ending dissociation with fetal calf serum (FCS), SC were seeded in FN-coated flasks, and expanded in Dulbecco’s modified Eagle medium, containing 10% heat-inactivated FCS serum, penicillin (100 mg/ml), streptomycin (100 U/ml), human recombinant Neu-differentiation factor ß (hrNDFß) (125 ng/ml), insulin (10 µg/ml) and forskolin (2 µg/ml). SC were purified by differential adhesion [37], and used at passage P2 or P3. Purification was controlled by immunocytochemistry for p75 and GFAP as markers of non-myelinating Schwann cells [33], and exclusion of the Thy1-2 marker of mouse fibroblasts [14]. For adhesion, migration and blocking receptor assays, SC were maintained in Sato serum-free medium [13] supplemented with hrNDFβ (125 ng/ml), and forskolin (2 µg/ml).

*iDISCO whole*-*mount immunofluorescence and imaging* Spinal cords were processed as described in the iDISCO protocol [52], including modifications described in the updated online protocol (https://idisco.info, Dec 2016). The primary antibody used was rabbit anti-RFP (1:1000, Rockland). Secondary antibodies used were donkey anti-rabbit Cy3 (1:800, Jackson Immunoresearch) and donkey anti-mouse IgG Cy5 (1:800, Jackson Immunoresearch) for intravascular staining. The cleared samples were imaged with a light sheet microscope (Ultramicroscope II; LaVision Biotec).

*RNA transcriptome analysis* For RNA preparations, SC from *Krox20*^*Cre/*+^*,R26*^*mT*/+^ (TdTomato) were freshly isolated from 2-week-old sciatic nerves. Nerves were cultured 4 days in vitro in the absence of growth factor to allow SC to de-differentiate and migrate out of the nerve. SC were then selected by FACS based on tdTomato expression. Library preparation and Illumina sequencing were performed at the IBENS genomic core facility. Briefly, (polyA +) mRNAs were purified from 250 ng of total RNA using oligo(dT). Libraries were prepared using the strand-specific RNA-Seq library preparation TruSeq Stranded mRNA kit (Illumina). Libraries were multiplexed by 6 in 1 high-output flow cells. A 75-bp read sequencing was performed on a NextSeq 500 device (Illumina). A mean of 94 ± 9.5 million reads passing Illumina quality filter was obtained for each of the six samples.

The analyses were performed using the Eoulsan pipeline [35], including read filtering, mapping, alignment filtering, read quantification, normalization and differential analysis. Before mapping, poly N read tails were trimmed, reads of ≤ 40 bases were removed, and reads with quality mean of ≤ 30 bases were discarded. Reads were then aligned against the Mus musculus genome from Ensembl version 84 using STAR [22]. Alignments from reads matching more than once on the reference genome were removed using the Java version of SamTools [39]. All overlapping regions between alignments and referenced gene were counted using HTSeq-count 0.5.3 [4]. The sample counts were normalized using DESeq 1.8.3 [3]. Statistical treatments and differential analyses were also performed using DESeq 1.8.3.

*Data availability* The RNASeq gene expression data and raw fastq files are available on the GEO repository (www.ncbi.nlm.nih.gov/geo/) under accession number: GSE107401 (accession password: mlkfkwoezxujvsn).

*Myelin protein extract isolation* Myelin was purified by sucrose gradient centrifugation [48]. Cerebral hemispheres of adult mice (3 months old) were homogenized on ice in 0.35 M sucrose and 5 mM EGTA, and the suspension was overlaid onto an equivalent volume of 0.85 M sucrose and 5 mM EGTA, and centrifuged at 100,000×*g* at 4 °C for 20 min. The myelin-containing fraction at the interface was collected, diluted threefold in distilled water, and centrifuged at 100,000×*g* at 4 °C for 30 min. After washing with distilled water, the isolated myelin pellet was resuspended in 20 mM Tris–HCl, aliquoted, and stored at – 20 °C.

*Pre*-*clustering of recombinant EphrinB3*–*Fc* Mouse EphrinB3–Fc fragments and human Fc were purchased from R&D Systems. The soluble forms of EphrinB3–Fc and its control Fc have low effect on receptor activation [19]; therefore, they were mixed with anti-mouse Fc–IgG and anti-human Fc–IgG (Alexa 555), respectively (ratio = 1:5), and incubated for 1 h at 37 °C prior to addition to SC [25].

*Adhesion and spreading assays* Adhesion and spreading in vitro assays were performed in 24-well dishes. Silicon strips on coverslips were used to separate two coated areas of each coverslip [8]. Surfaces were coated overnight at 37 °C with recombinant EphrinB3–Fc fusion at 10 µg/mL and Fc equimolar (as control) on each half, or myelin extract (100 µg/mL) and PBS buffer (as control). Before cell seeding, strips were removed and coverslips were washed carefully with PBS. 10^5^ SC were seeded in serum-free Sato medium to avoid proliferation, and allowed to adhere for 3 h. Data were always expressed as ratio in respect to the intra-coverslip control [12].

*Survival assay* GFP^+^SC were seeded on uncoated glass coverslips in normal medium. After overnight adhesion, medium was changed, adding Sato serum-free medium supplemented with clustered EphrinB3 at 10 µg/mL or Fc equimolar (as control), or with myelin extract (100 µg/mL) or PBS (as control). SC were incubated for 3 h or 24 h as specified in each experiment. After fixation in 4% paraformaldehyde (5 min), SC were immuno-stained for caspase 3 adding Hoechst dye to visualize all nuclei, and coverslips were mounted with fluoromount.

*Migration assay* SC were resuspended at 3 × 10^6^ cells/ml in Sato medium containing 0.8% low-melting point agarose (Sigma). One drop (1.5 μL) of this suspension was applied to the center of FN +EphrinB3, or FN +Fc-coated glass coverslips, which were placed at 4 °C for 1 min to allow the agarose to solidify. The cooled drop was covered with Sato medium with hrNDFß (125 ng/ml) and forskolin (2 µg/ml), and placed up to 6 h at 37 °C in the incubating chamber of a video-microscope (ZEISS).

*SC receptor blocking assay* EphA4 and EphB6 receptors or Integrinβ1 were neutralized in SC by incubation with anti-EphA4 (1.2 µg/10.000 cells, R&D, AF641), anti-EphB6 (1.2 µg/10.000 cells, Santa Cruz Biotechnology, sc-7282), anti-integrinβ1 (0.6 µg/10.000 cells, MA2910, Thermo Fisher Scientific) antibodies or IgG (as control) in Sato medium for 1 h at 37 °C prior to cell seeding or transplantation.

*Immuno*-*staining* Cultured SC were fixed for 5 min in 4% paraformaldehyde prior to immuno-staining and mice were killed by trans-cardiac perfusion of PBS followed by cold 4% paraformaldehyde, and post-fixed in the same fixative for 1 h. Spinal cords were cryo-protected by immersion in 20% sucrose solution overnight, embedded in cryomatrix (Thermo Scientific), and frozen in cold isopentane at − 60 °C. Finally, they were sectioned with a cryostat at 12 µm (Leica Microsystems). Both cells and sections were washed, blocked in 5% BSA for 40 min and incubated with the primary antibodies. While cells were incubated 1 h at room temperature, slides were incubated overnight at 4 °C. For MOG staining, sections were incubated with absolute ethanol for 10 min followed by primary antibody, and then washed profusely. Primary antibodies were as follows: anti-EphA4 (1:50, AF641, R&D Systems); anti-EphA4-Tyr(602) (1:50, EP2731, ECM Biosciences); anti-EphB6 (1:50, SAB4503476, Sigma); anti-EphB1 (1:50, SAB4500776, Sigma; anti-Eph receptor B1 + Eph receptor B2 (phospho Y594) (1:50, ab61791, Abcam); anti-Ki67 (1:100, 556003, BD Biosciences); anti-cleaved caspase3 (1:500, 9661S, Cell Signalling); anti-GFP (1:400, GFP-1020, Aves); anti-MOG (1:20, mouse IgG1 hybridoma, clone C18C5; provided by C. Linnington, University of Glasgow, Glasgow, United Kingdom); anti-MBP (1:50, ab7349, Sigma); anti-Glut1 (1:100, 07-1401, Merck Millipore; and 1:400, MABS132, Sigma); anti-Fibronectin (1:600, F6140, Sigma); anti-CD31 (1:200, 553370, BD Pharmigen); anti-NF200 (1:200, N4142,Sigma), anti-p75 (1:100, 8238S, Ozyme), anti-CD13 (1:50, MCA2183, BioRad), anti-CD68 (1:400, MCA1957, BioRad), anti-CD11b (1:400, MCA74G, BioRad), anti-F8/40 (1:100, MCA497R, BioRad), anti-Collagen 4(1:400, ab19808, Abcam), anti-Olig2 (1:300, MABN50, Millipore); anti-sox10 (1:50, AF2864, R&D Systems); and anti-CD13 (1:200, 553370, BD Pharmingen). Next, cells or sections were washed and incubated with secondary antibodies and Hoechst dye for 1 h at room temperature. The excess secondary antibody was removed by several PBS washes, and coverslips/slides were mounted using fluoromount.

*Electron microscopy* For electron microscopy, mice were perfused with PBS followed by 4% paraformaldehyde/2.5% glutaraldehyde (Electron Microscopy Science) in PBS for 45 min. Dissected spinal cords were post-fixed with the same solution for 2 h, then sectioned into 60-µm slices with a vibratome and washed twice with PBS before enzyme immunolabeling. For DAB revelation, endogenous peroxidase was inhibited with a methanol/oxygen peroxide incubation, and washed and blocked by 5% BSA for 1 h. Sections were incubated with anti-GFP overnight at 4 °C, then washed and incubated with a secondary biotinylated antibody for 2 h at room temperature. After several washes with PB 0.1 M, sections were incubated with the ABC kit (VECTASTAIN^®^ ABC-HRP Kit, Vector Lab) containing peroxidase–anti-peroxidase for 40 min followed by a DAB/oxygen peroxide mix before stopping the reaction with distilled water. Samples were fixed in 2% osmium tetroxide (Sigma-Aldrich) 30 min, washed gently and incubated with 5% uranyl acetate for 30 min in the dark. After dehydration, samples were embedded in Epon resin 812. Ultra-thin sections (80 nm) were examined with a HITACHI 120 kV HT-7700 electron microscope.

*Demyelinating lesions and grafts* Wild-type mice were anaesthetized with a ketamine/xylazine mixture. Demyelination was induced by stereotaxic injection of lysolecithin (LPC) (1%, 0.5 µl) in PBS. LPC or PBS (in control animals) was injected into the dorsal funiculus of the spinal cord at the level of T8–T9 in the dorsal column of white matter using a glass micropipette. SC (10^5^/2 µL) were injected the same day, two vertebrates caudally (4 mm) in the same tract.

*Western blotting* SC (3 × 10^5^ cells/well) were lysed in RIPA buffer with Complete^®^ and Phosphostop^®^ inhibitor, and analyzed by electrophoresis in an SDS 4–20% MINI PROTEAN TGX gel. After electrophoresis, proteins were transferred electrophoretically to polyvinylidene difluoride membranes and probed with the following antibodies:anti-EphB6 (1:500, SAB4503476, Sigma), anti-EphB1 (1:500, SAB4500776, Sigma), anti-EphA4 (4 µg/mL, 37-1600, ThermoFisher), anti-p-EphB1 + 2 (1:300, ab61791, Abcam), anti-p-EphA4 (1:300, EP2731, ECM Biosciences), anti-Integrinβ1 (1:500, 550531, BD Pharmingen), anti-EphrinB3 (1:250, 1 µg/mL, AF395 R&D Systems), anti-MBP (1:1000, ab980, Millipore), anti-GAPDH (1:5000, MAB374, Millipore) and anti-Actin (1:50,000, A2228, Sigma). Peroxidase-conjugated anti-rabbit, anti-goat or anti-mouse IgG secondary antibodies (Jackson Immuno Research) were used at a dilution of 1:5000, 1:10,000 and 1:20,000, respectively, and anti-Rat biotinylated (1:100, Vector Labs) followed by peroxidase-conjugated streptavidin. Protein bands were visualized by chemoluminescence (ECL BioRad). Intensity of the bands was quantified with FIJI.

*Neutralization of EphrinB3 epitopes in myelin extracts* EphrinB3 epitopes in myelin extract proteins were neutralized by incubation with anti-EphrinB3 antibodies (AF395, R&D system and sc-271328, Santa Cruz Biotechnology, ratio: 1:1) for 2 h at room temperature prior to the addition to the cells [55].

*Spinal cord live imaging* LPC lesion followed by GFP^ + ^SC engraftment was performed in 60-day-old mice and terminally anesthetized for imaging 36 h later. Rhodamine-labeled BSL I (Vector Labs RL-1102) was injected at 2 mg/ml in the beating heart to label BV. After 5 min allowing dye circulation, spinal cords were dissected in ice-cold HBSS solution supplemented with 6.4 mg/mL D-(+)-glucose and bubbled for 30 min with bubbled with 95% O_2_/5% CO_2_. Spinal cord segments including lesion and graft sites were laid onto Millicell-CM slice culture inserts (Millipore) over culture medium (50% DMEM + Glutamax, 25% HBSS, 25% heat-inactivated horse serum, 5 mg/mL D-(+)-glucose, 20 mM Hepes, penicillin (100 mg/ml), streptomycin (100 U/ml), hrNDFß (125 ng/ml), and forskolin (2 µg/ml) in glass bottom plates, and then placed in an inverted Leica SP8X confocal microscope with an on-stage incubator, while streaming 95% O_2_ and 5% CO_2_ into the chamber. Spinal cords were imaged using a 25 × immersion objective at intervals of 15 min during 12 h with intermittent repositioning of the focal planes. Maximum intensity projections of the collected stacks (~ 60 μm at 2 μm step size) were compiled in FIJI.

### Quantification

*Lesions* The lesion area was identified by immune detection of GFAP combined with Hoechst^+^-labeled nuclei to reveal astrocyte reactivity and hyper-cellularity, respectively.

*SC adhesion and spreading* In vitro SC adhesion on different surfaces was quantified as the ratio of the number of adhered GFP^+^ on myelin- or EphrinB3-coated areas, over those adhered to uncoated or Fc-coated area within the same coverslip. All coated areas were of equal size. SC spreading was evaluated by quantifying the ratio of “round” GFP^+^SC (lacking processes) out of the total number of cells on myelin- or EphrinB3-coated areas over those on non-coated or Fc coated areas. “Round cells” were defined as cells with no processes at all, thus with a perfectly circular shape.

*In vitroSC extent of migration* SC migration was quantified by measuring the number of SC outside the 1.5-µL agarose drop and the maximum extent of their migration from the edge drop.

*In vitroSC velocity* SC speed of migration was quantified by manual cell tracking plugging in FIJI, calibrating pixel size and duration of time lapse of each frame.

*Size of lesion and graft area* Lesion and grafted cells within the dorsal funiculus were quantified by delimiting Hoechst ^+^ nuclei hyper-density and GFAP-positive area on 12-µm section. Lesion and graft areas were quantified by ImageJ 1.49 s. For each animal, at least three serial sections with 60-µm intervals were quantified.

*In vivoextent of SC migration* SC migration within the dorsal funiculus was quantified on longitudinal sections evaluating the distance between the graft injection site and the most proximal GFP^+^ cell to the lesion (LPC injection site) in each animal from the different groups.

*SC*–*BV association* SC–BV association was quantified in the intermediate zone, at 1 mm from the graft edge in the direction of the lesion or in the lesion site. GFP^+^/Hoechst^+^ SC, with the whole or more than half of the cell area in contact with Glut1^+^ endothelial cells, were counted as “closely associated cells” while those with only “tip” contacts or no contacts were considered as “not associated cells”. Data are expressed as the percentage of total counted cells in both groups.

*SC*–*axon alignment in the lesion*. GFP^+^/Hoechst^+^ SC, with the whole or more than half of the cell in parallel orientation and aligned closely to NF200^+^ axons (excluding alignment to BV), were counted as “closely associated cells” while those with only “tip” contacts or no contacts were considered as “not associated cells”. Data are expressed as the percentage of total counted cells in both groups.

### Statistics

The sample size calculation was performed by the resource equation method to minimize the sample size, following the ARRIVE guidelines for reporting animal research. Each n represents one animal or SC sample in the experiment. The grafting experiments were repeated at least three times with a different set of animals each. For the in vitro analysis, experiments were performed at least three times with SC obtained from different dissections and dissociations. Statistical analysis was carried out using GraphPad Prism 6 software. All values were expressed as mean ± SD. Normality in the variable distributions was assessed by the D’Agostino–Pearson omnibus test and Grubbs’ test was used to detect and exclude possible outliers. When normality test was passed, means were compared by two-tailed Student’s *t* test. When one or both groups did not follow a normal distribution, means were compared by two-tailed Mann–Whitney *U* test. When different independent groups were compared, we performed a one-way ANOVA plus Tukey’s multiple comparison tests. One-sample *t* test was used to compare values to the hypothetical mean: 1 for ratios and 100 for percentages. Repeated measure ANOVA was used to analyze the difference along time of a certain parameter. *P*-values lower than 0.05 were used as a cut-off for statistical significance.

## Results

### Schwann cells’ preferential migration along blood vessels bypasses their inhibition by myelin

**Fig. 1:**
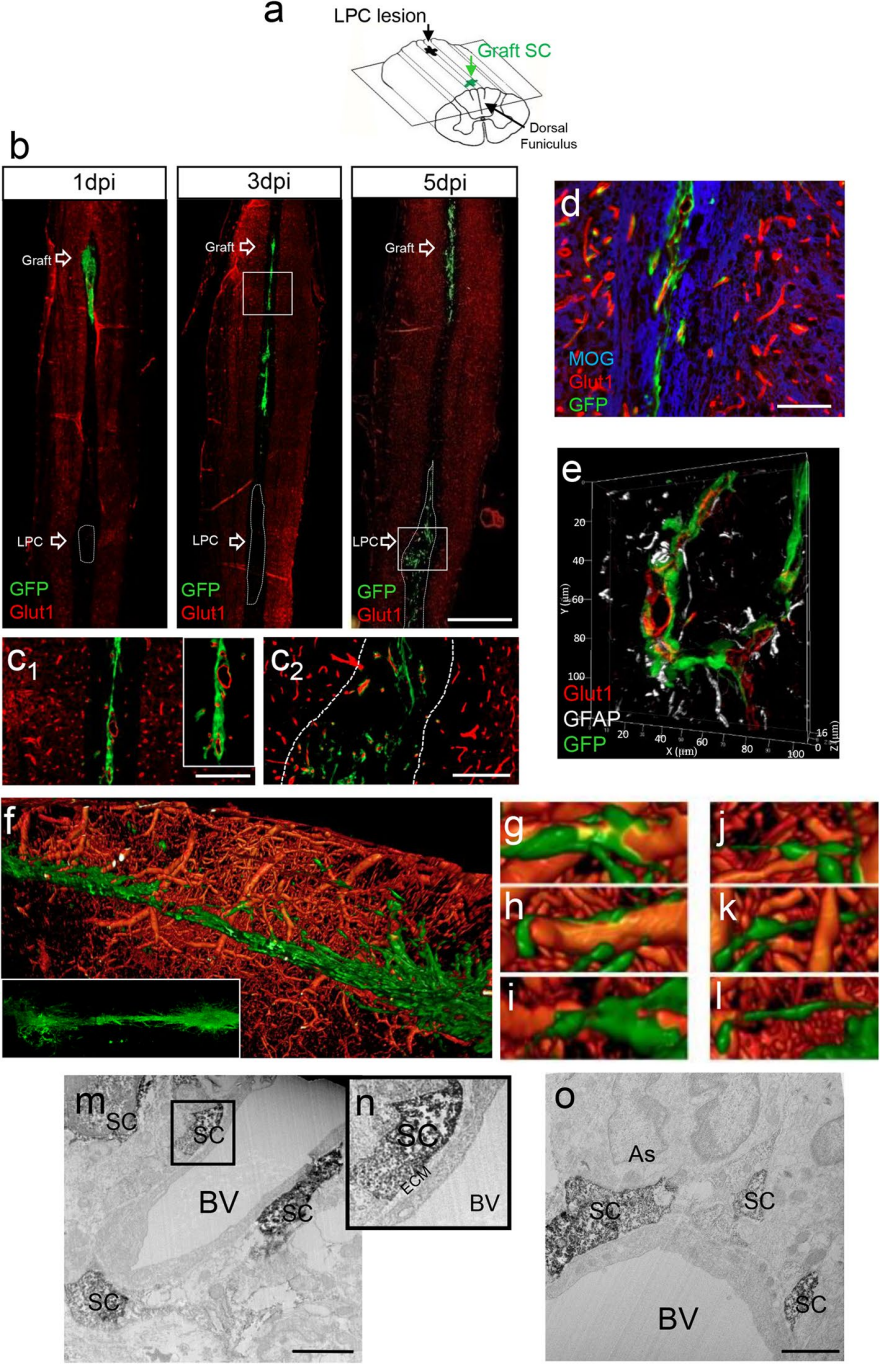
Migrating behavior of SC grafted in white matter remotely from LPC-induced demyelination. **a** Scheme of LPC lesion and SC graft targeted into the dorsal funiculus of the spinal cord. Graft and lesion are 4 mm apart. **b** General view of longitudinal sections of the spinal cord illustrating the graft and lesion sites at 1 dpi, 3 dpi and 5 dpi, scale bar 1000 µm. **c**_**1**_–**c**_**2**_ GFP^+^SC grafted within dorsal spinal cord white matter migrate preferentially in close contact with Glut1^+^ endothelial cells when progressing along the midline, towards the lesion (**c**_**1**_) and spreading within the lesion (**c**_**2**_). Dotted lines identify the lesion sites, scale bar 200 µm. **d** Grafted GFP^+^SC along the midline avoid MOG^+^ myelin and are associated with Glut1^+^BV, scale bar 100 µm. **e** 3D Z stack reconstruction illustrating GFP^+^SC located between Glut1^+^endothelial cells and GFAP + perivascular astroglial end-feet. **f**–**l** 3D reconstruction after light sheet imaging of clarified whole spinal cord illustrating in (**f**) the abundant vascular network and tdTomato^+^SC migrating from the graft along the dorsal funiculus midline en route toward the lesion. **g**–**l** Most tdTomato^+^SC exiting the graft are polarized on BV (**g**–**i**) or between BV-evoking jumping events (**j**–**l**). TdTomato given color code is green, and BV is red. **m-o** Immuno-EM of GFP^+^SC in the midline illustrates several GFP^+^SC revealed by DAB embedded in perivascular ECM (**m**) and between BV and astrocytes (As) (**o**), scale bar 2 µm. **n** Higher magnification of the boxed area in (**m**)

To explore how endogenous SC invade the CNS in response to demyelination, we used the Krox20Cre driver line crossed over Rosa–YFP to track the SC lineage [18, 44]. Krox20 transcription factor is specific to the PNS, and is expressed in the SC lineage until adulthood. LPC injections were performed in the dorsal funiculus of adult mice (2–3 months old), and mice were killed at 3 days post-injection (dpi). YFP^+^SC were detected only in the spinal cord cross sections of demyelinated mice (Suppl. Fig. 1c,d) but not in controls (Suppl. Fig. 1a, b). YFP + cells were Sox10^+^ (Suppl. Fig. 1e,f) but never Olig2 + (Suppl. Fig. 1g) validating their SC lineage. We next examined the location of the SC-derived population and found that at this early time point, the YFP^+/^Sox10^+^SC within white matter were found exclusively on Glut1^+^BV. Glut1 specificity was assessed by co-immunolabeling with different lineage markers. Glut1 expression co-localized with the endothelial marker CD31 ([16]; Suppl. Fig. 2a-c) but not with the pan microglial/macrophage markers CD68, CD11b, and F8/40 (Suppl. Fig. 2d–f), the SC precursor and non-myelinating SC marker p75 (Suppl. Fig. 2g–i), the pericyte marker CD13 (Suppl. Fig. 2j–l) and the SC/astrocyte marker GFAP (Fig. 1e), thus confirming the specificity of Glut1 for endothelial cells.

The location of SC indicates that endogenous SC preferentially associate with BV when triggered to invade the demyelinating CNS. Although Krox20 specifically labels the SC lineage in normal development, Krox20 was also expressed by Iba1^ +^ microglial cells under pathological conditions (Suppl. Fig. 1h, i).

As the Krox20^Cre^ system was limited in tracing a sufficient number of SC migrating within the CNS, we pursued our investigation using an exogenous paradigm in which GFP^+^ SC were grafted two vertebrates away from a LPC lesion in the dorsal funiculus of adult wild-type mice (Fig. 1a). In this paradigm, grafted SC are known to travel long distances (up to 2 mm) before being recruited specifically by the lesion, modeling SC recruitment after CNS injury, whereas they remain at the graft site in the absence of a lesion [7, 17]. As our study focused essentially on SC migration/recruitment, grafted animals were killed at early time points including 1, 3 and 5 dpi. The spatio-temporal distribution of the transplanted GFP^+^ SC was assessed by scanning longitudinal frozen sections of the spinal cord for the GFP signal throughout the sections [17]. At 1 dpi, lesion size (0.40 ± 0.06 mm^2^ per section) and GFP^+^SC-grafted area (0.16 ± 0.08 mm^2^ per section) showed minor variability among animals validating the lesion–graft paradigm. Analysis of the GFP signal over time confirmed the spatio-temporal progression of SC towards the lesion, which was systematically reached at 5 dpi (Table 1, Fig. 1b).

**Table 1:** Time regulation of SC arrival at the lesion

	Distance graft–LPC injections (mm)	Distance SC towards lesion (mm)	Animals with SC within the lesion (%)
1 dpi	4.3 ± 0.44	1.06 ± 0.48	0
3 dpi	4.5 ± 0.44	2.75 ± 0.88	28
5 dpi	4.1 ± 0.69	4.57 ± 0.79	100

Extent of migration and percentage of animals with lesions containing SC at different times. Data are expressed as mean ± SD at 1 dpi (*n* = 5), 3 dpi (*n* = 6), and 5 dpi (*n* = 7)

In the intermediate zone connecting the graft to the lesion, GFP^+^SC migrated preferentially along the midline avoiding myelin (Fig. 1b, c_1_, d), as previously described after engraftment in shiverer and nude mice [7, 17]. In this area, SC were found preferentially in association with Glut1^+^BV forming a narrow stream of cells (Fig. 1c_1_, d). At their arrival in the lesion, GFP^+^SC were no longer confined to that narrow path, but randomly spread within the lesion. Co-detection of GFP and Glut1 at the lesion showed that the pattern of GFP^+^SC matched with that of Glut1^+^BV (Fig. 1c_2_). Co-labeling for Glut1 and GFAP showed that GFP^+^SC were localized in perivascular spaces between endothelial cells and astrocyte end-feet (Fig. 1e). SC within the BV lumen were never observed.

We used whole-mount immunolabeling of clarified spinal cords to gain insight in SC–BV 3D spatial organization. For these experiments, tdTomato^+^ SC were grafted remotely from the lesion as above, and their migratory behavior was analyzed at 5 dpi. BV and SC were visualized by immunolabeling using anti-IgG and anti-Tomato, respectively. Light sheet imaging and 3D reconstruction revealed the dense spinal cord vascular network and confirmed that grafted SC, en route towards the lesion, were preferentially associated with BV in the spinal cord midline (Fig. 1f). TdTomato^+^ SC were either polarized along blood vessels (Fig. 1g–i) or extending processes from one vessel to another (Fig. 1j–l).

That SC move along blood vessels was further confirmed by GFP^+^ SC live-imaging 2 dpi in the demyelinated spinal cords and host BV were labeled by intra-cardiac perfusion of rhodamine-lectin. Spinal cord whole mounts were maintained in appropriate physiological conditions, and areas containing GFP^+^SC were selected and video-recorded for 20 h. Recordings confirmed that SC leaving the graft reached for and associated with BV, to migrate either in chains or as isolated cells, moving from one BV to the next (MovieS1, Suppl. Fig. 3a, blue empty arrowhead) or sliding along them (MovieS1, Suppl. Fig. 3a, white arrowhead) as described above. In the chains of migrating cells, individual cells could break off from the chain and migrate independently, later rejoining a second chain (Movie S2, Suppl. Fig. 3b arrowhead).

To elucidate whether SC migrate in vivo in association with perivascular ECM, and/or whether they require direct interactions with endothelial cells, we performed immuno-electron microscopy (EM) on a novel series of GFP^+^SC-grafted mice (Fig. 1m–o). EM analysis of DAB-labeled GFP^+^SC confirmed their location in close contact with BV. Interestingly, the majority of the grafted SC was embedded within the perivascular ECM (Fig. 1m, n) between the vascular cells and astrocyte end-feet (Fig. 1o). However, unlike in the injured PNS [16], direct contact with vascular cells was never observed.

**Fig. 2:**
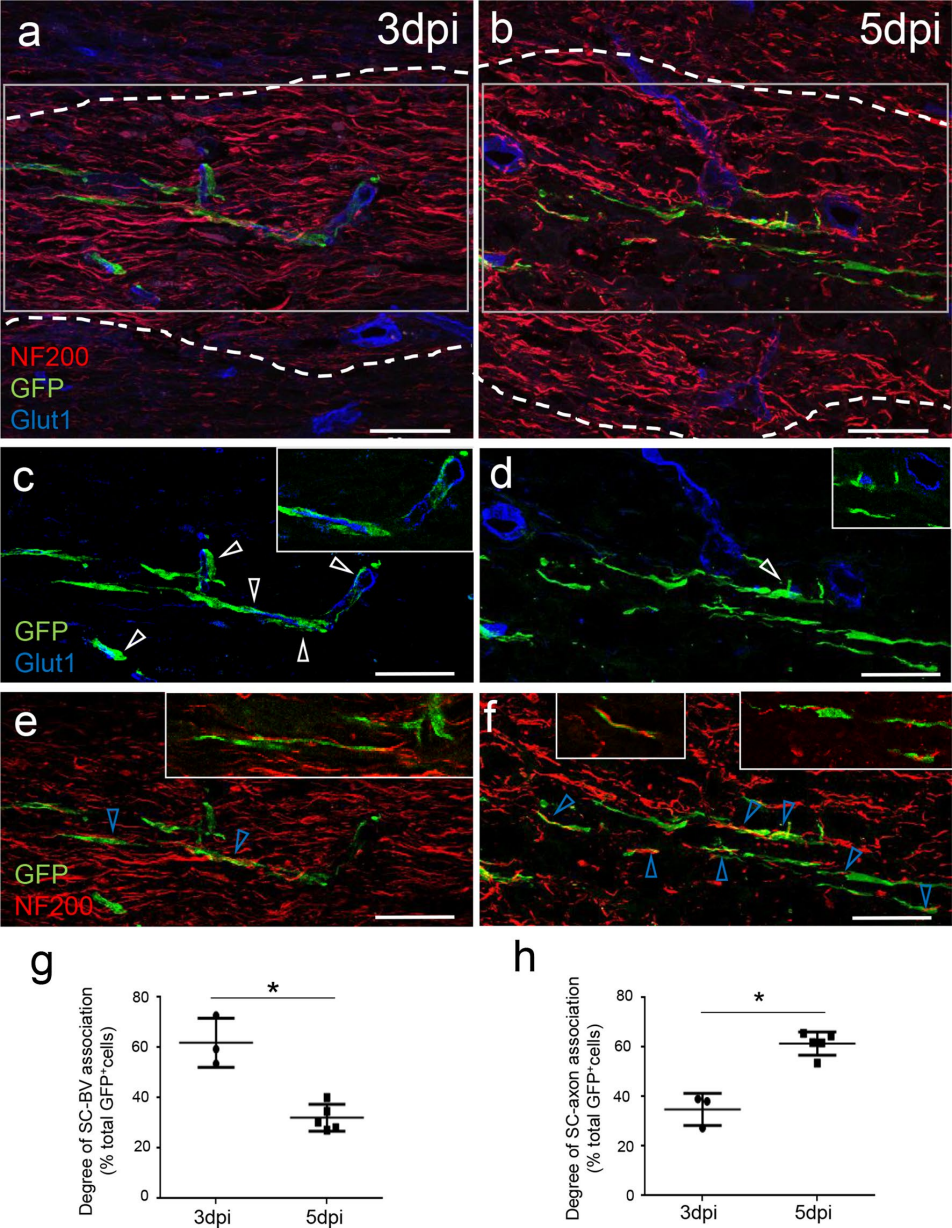
SC arrive at the lesion along BV and timely dissociate from them to contact demyelinated axons. **a**, **c**, **e** Upon arrival at the lesion at 3 dpi, grafted GFP^+^SC are associated with BV. **b**, **d**, **f** In lesions at 5 dpi, fewer SC are associated with BV but are aligned with NF200^+^axons. **c**–**f** Higher magnifications of GFP^+^ SC illustrating the temporal decrease of association of GFP^+^SC with Glut1-positive BV (white arrows) (**c**, **d**) compared to the progressive increase of GFP^+^SC association with NF200^+^ axons (blue arrow) (**e**–**f**). **c**, **e** and **d**, **f** show separated colors of **a** and **b**, respectively. Images represent confocal maximal projections of Z-stacks, while insets show only one confocal Z-plane. Scale bar 50 µm. **g**, **h** Quantification of SC associated or not with BV at 3 dpi (*n *= 2) and 5 dpi dpi (*n *= 8) (two-tailed Mann–Whitney test *p *= 0.035) (mean value ± SD of three independent experiments)

Temporal analysis of SC arrival at the lesion indicated that when cells were present at early stages of the lesion (3 dpi), 62 ± 9% of GFP^+^SC were closely associated with BV (Fig. 2a, c, g), while at later times (5 dpi) only 32 ± 5% were associated with BV (Fig. 2b, d, g). Immunohistochemistry for Glut1 and NF200 indicated that this transition correlated with a change in SC–BV to SC–axon associations, with only 35 ± 6% of GFP^+^SC associated with axons at 3 dpi (Fig. 2a, e, h), and 61 ± 5% GFP^+^SC associated with axons at 5 dpi (Fig. 2b, f, h).

### EphrinB3 present in myelin is able to bind and activate EphrinB3 receptors in Schwann cells

We previously demonstrated that SC are repulsed by CNS myelin, partially mediated by the axonal-repellent MAG, suggesting that other myelin components might be involved in this repulsion [17]. Therefore, we hypothesized that SC guidance within the CNS by SC–BV association and myelin repulsion could be due to some receptor–ligand-mediated mechanism. Thus, we speculated that other axonal growth inhibitors were able to induce the same effect in SC. Of interest, EphrinB3 is expressed by CNS myelin and inhibits axonal growth and oligodendrocyte differentiation [23, 55].

**Fig. 3:**
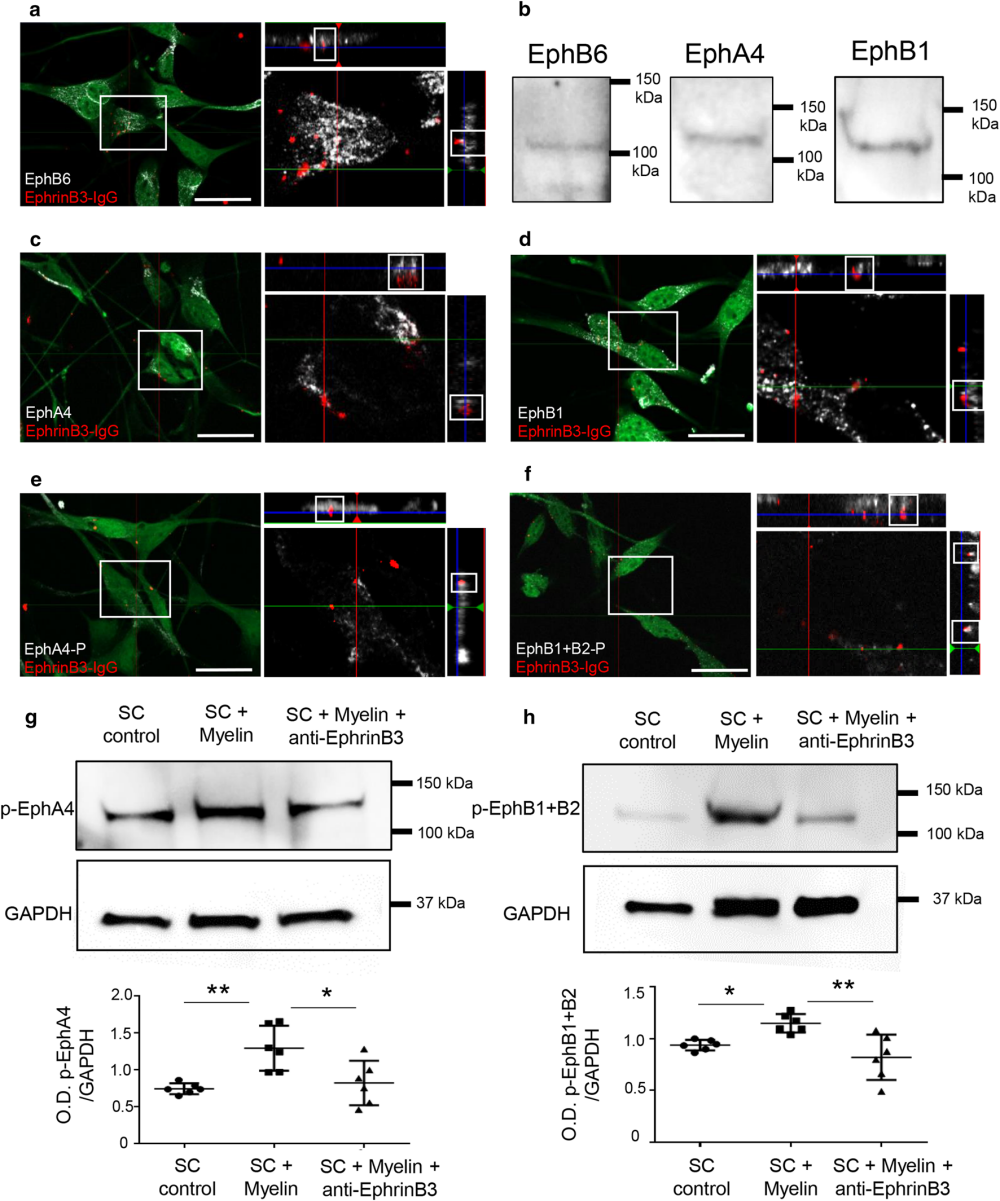
EphrinB3 binds and activates EphA4, EphB1 and EphB6 receptors in SC. **a**, **c**, **d** Orthogonal views of SC activation with clustered EphrinB3 shows that EphrinB3 binds EphB6 (**a**), EphA4 (**c**) and EphB1 (**d**) receptors on SC. **b** Western blot confirming the presence of these receptors in SC. **e**, **f** Bound EphrinB3 activation of EphA4 and EphB receptors viewed by immunodetection of phosphorylated forms. **g**, **h** Western blot illustrating that SC incubation with myelin increased the phosphorylation of EphA4 (**g**) and EphB1 + B2 (**h**) which was not the case when myelin was previously blocked by anti-EphrinB3 (O.D. p-EphB1 + B2/GAPDH: one-way ANOVA *p *= 0.003, F(2,15) = 8.57, followed by a Tukey’s multiple comparison test) and O.D. of p-EphA4/GAPDH: one-way ANOVA *p *= 0.0036, F(2,15) = 8.44, followed by a Tukey’s multiple comparison test). Data are expressed as ratio of the optical density (O.D.) of the bands (mean values ± SD) from three independent experiments, control (*n *= 6), myelin (*n* = 6), myelin + anti-EphrinB3 (*n *= 6). Scale bar 20 µm

We first confirmed that mouse SC have the molecular machinery to bind myelin-associated EphrinB3. Gene profiling by RNA sequencing revealed the expression of EphrinB3 receptors in purified mouse SC (Table 2) as previously observed in rat Schwann cells (1) [16]. Moreover, immunohistochemistry showed that these SC can bind EphrinB3 in vitro, and express three EphrinB3 receptors: EphB6 (Fig. 3a), EphA4 (Fig. 3c) and EphB1 (Fig. 3d). The presence of each receptor was corroborated by western blot (Fig. 3b). Incubation of SC with pre-clustered EphrinB3 by fluorescent anti-Ig and orthogonal views revealed that the three receptors were able to bind clustered EphrinB3 on SC surface leading to the formation of large signaling clusters as previously described [56] (Fig. 3a, c, d).

**Table 2:** RNA sequencing analysis

Id	Associated gene name	Mean_WT	Description
ENSMUSG00000026235	EphA4	755,71	Eph receptor A4 [Source:MGI Symbol;Acc:MGl: 98277]
ENSMUSG00000032537	EphB1	274,75	Eph receptor B1 [Source:MGI Symbol;Acc:MGl: 1096337]
ENSMUSG00000028664	EphB2	1909,59	Eph receptor B2 [Source:MGI Symbol;Acc:MGl: 99611]
ENSMUSG00000005958	EphB3	2194,48	Eph receptor B3 [Source:MGI Symbol;Acc:MGl: 104770]
ENSMUSG00000029710	EphB4	3541,54	Eph receptor B4 [Source:MGI Symbol;Acc:MGl: 104757]
ENSMUSG00000029869	EphB6	7149,84	Eph receptor B6 [Source:MGI Symbol;Acc:MGl: 1096338]

SC expression profiles of Eph receptors, *n* = 3

Eph receptors are activated by auto-phosphorylation of specific tyrosine residues [42]. To verify the ability of EphrinB3 to induce forward signaling, activation of Eph receptors on SC was assessed. Immunocytochemistry illustrates binding of EphA4 with clustered EphrinB3, and its phosphorylation by the ligand (Fig. 3e). Immunohistochemistry also validated the presence of phosphorylation in EphB1 (and EphB2) receptor, and its interaction with bound EphrinB3 in SC (Fig. 3f), which can undergo trans-phosphorylation by the kinase-defective receptor EphB6 [27].

To test the hypothesis that myelin-associated EphrinB3 is able to activate EphB1 + B2 and EphA4 receptors, we incubated purified SC during 30 min with myelin protein extracts in the presence or not of anti-EphrinB3 to specifically block its activity [55]. SC extracts were blotted with either an antibody against phosphorylated EphA4 (Fig. 3g) or an antibody against phosphorylated EphB1 + B2 (Fig. 3h). Myelin extracts significantly increased the phosphorylated forms of both receptors compared to the housekeeping GAPDH. However, no signal increase was observed when myelin extracts were pre-incubated with anti-EphrinB3 antibody (O.D. of p-EphB1 + B2/GAPDH control: 0.93  ±  0.05, myelin: 1.1 ± 0.08, myelin + anti-EphrinB3: 0.82 ± 0.21) and O.D. of p-EphA4/GAPDH control: 0.74 ± 0.07, myelin: 1.29 ± 0.30, myelin + anti-EphrinB3: 0.82 ± 0.30). Thus, blocking the epitopes of EphrinB3 in myelin neutralized the Eph receptor activation by myelin on SC (Fig. 3g, h).

Collectively, these observations evidence the ability of SC to bind and respond to the presence of EphrinB3 in vitro.

### CNS myelin inhibits Schwann cell adhesion and spreading, and this effect is partially mediated by EphrinB3 through EphA4 and EphB6 receptors

**Fig. 4:**
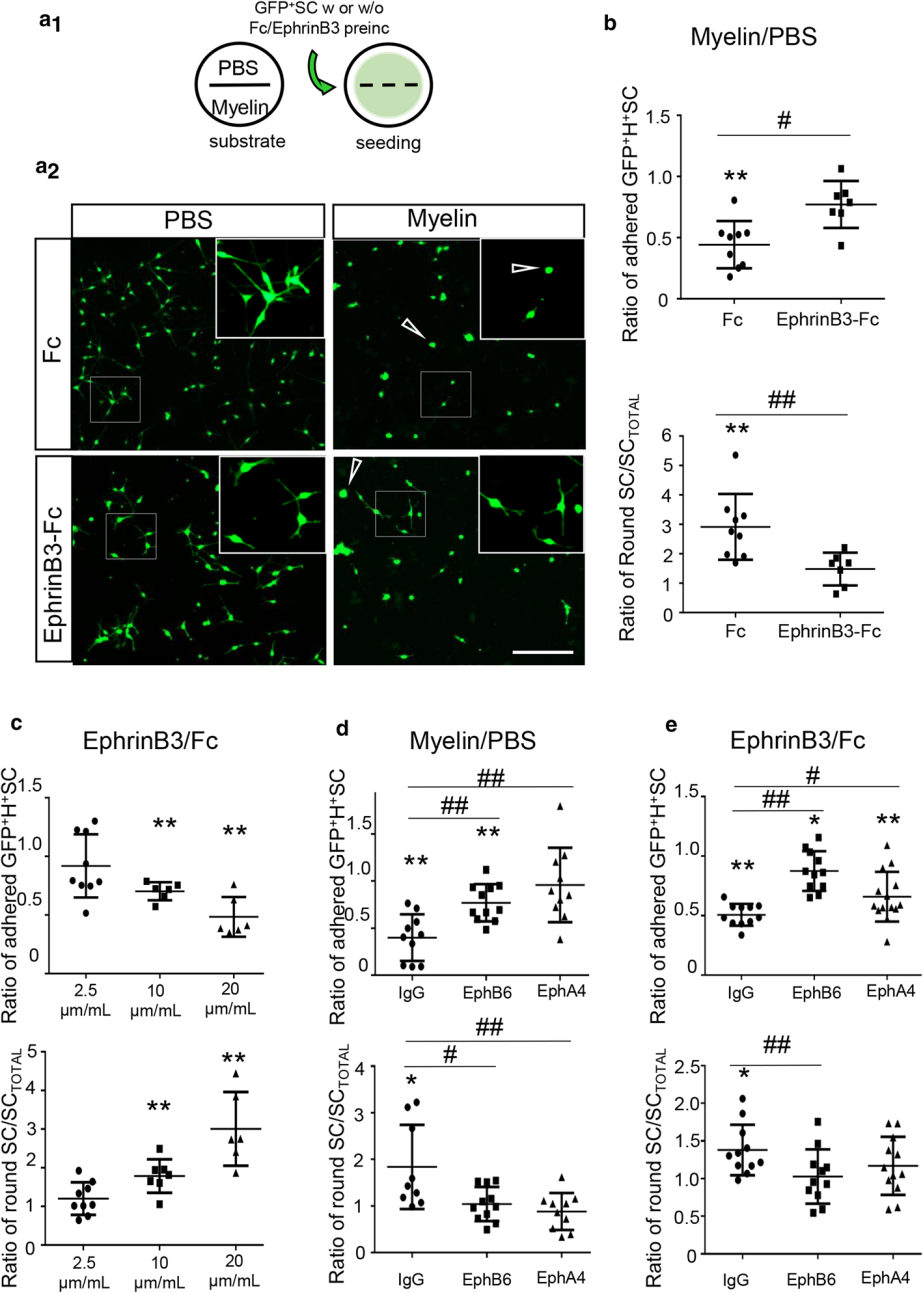
EphrinB3 mediates myelin inhibition on SC adhesion and polarization through EphA4 and EphB6 receptors. **a**_**1**_ Diagram of the adhesion assay. Coverslips divided by silicon strips were coated with PBS or myelin on each half. After strip removal, SC pre-incubated with Fc or EphrinB3 were seeded homogenously on the coverslip. **a**_**2**_ Unclustered EphrinB3 partially reverted myelin-induced inhibition of SC adhesion and spreading. Arrows show examples of “round cells”, scale bar 200 µm. **b** Quantification of the ratio of adhered or round GFP^+^SC on Myelin over PBS after pre-incubation with unclustered EphrinB3 or Fc. **c** SC seeded on substrate coated with increased concentrations of EphrinB3, adhered and spread less in a dose-dependent manner with respect to the intra-coverslip control substrate (Fc). Concentrations of EphrinB3 and Fc were 2.5 µg/mL, 10 µg/mL, and 20 µg/mL. **d**, **e** Quantification of the number of adhered and polarized SC pre-incubated with anti-EphB6 or anti-EphA4 (extracellular domain). Pre-incubation with anti-EphB6 and anti-EphA4 improved the number of adhered and polarized SC on myelin (**d**) and EphrinB3 (**e**) compared to PBS and Fc, respectively. Pre-incubation with IgG as control showed similar results as non-pre-incubated cells (**b**, **c**). Data are expressed as ratios (mean values ± SD from three independent experiments) of myelin or EphrinB3 surfaces compared to intra-coverslips control non-coated surfaces (PBS) or coated with equimolar concentration of Fc, respectively. In **b**, **c**, **d**, **e** */** are used for comparison of a group with a hypothetical mean of 1 by one-sample two-tailed *t* test, and #/## for comparison between two different groups by two-tailed Mann–Whitney test (*n *= 7–11 per group). */^#^*p *< 0.05; **/^##^*p *< 0.01

Next, we examined whether EphrinB3 could contribute to SC–myelin repulsion. We performed a blocking receptor assay, pre-incubating purified GFP^+^SC with unclustered EphrinB3-Fc molecules before seeding them for 3 h onto myelin protein extract or PBS (Fig. 4a), and evaluated both SC adherence and polarization (Fig. 4b). Control Fc-pre-incubated SC showed less adhesion to myelin, compared to SC adhering to PBS-coated surfaces. SC also extended fewer processes, based on a higher ratio of round cells over the total adhered SC on myelin compared to on PBS. Blocking EphrinB3 receptors on SC by soluble EphrinB3–Fc hindered myelin repulsion, resulting in a significant improvement of SC adhesion to myelin-coated surfaces, and increased ability to extend processes (fewer round cells) on myelin than those pre-incubated only with soluble Fc (ratio of adhered cells myelin/PBS: Fc-pre-incubated SC on myelin: 0.4 ± 0.2; EphrinB3-pre-incubated SC on myelin: 0.8 ± 0.2. Ratio of round cells myelin/PBS: Fc: 2.9 ± 1; EphrinB3: 1.5 ± 0.5).(Fig. 4a, b).

To determine if the reduction in the number of SC adhering to myelin was due to cell death, we assessed cell viability by comparing the percentage of caspase3^+^ nuclei or Hoechst^+^ pycnotic nuclei in SC incubated with PBS or myelin for 3 h. SC exhibited a typical bipolar morphology and no difference was observed in the percentage of caspase3^+^ nuclei or Hoechst^+^ pycnotic nuclei between control and myelin-treated groups (PBS: 4.4 ± 1.6‰, myelin: 4.0 ± 1.7‰, *n* = 6). These data corroborate our previous results demonstrating that short incubation time with myelin does not induce apoptosis [17].

SC adhesion to surfaces coated with EphrinB3–Fc was reduced compared to Fc as control in a dose-dependent manner (ratio of adhered cells EphrinB3–Fc: 2.5 µg/mL: 0.9 ± 0.3, 10 µg/mL: 0.7 ± 0.07 and 20 µg/mL: 0.5 ± 0.2. Ratio of round cells EphrinB3–Fc: 2.5 µg/mL: 1.2 ± 0.4, 10 µg/mL: 1.8 ± 0.4 and 20ug/mL: 3 ± 0.9) (Fig. 4c). Moreover, the increased number of round SC indicated that SC were less polarized on EphrinB3-coated surfaces compared to control (significance reached with high concentrations of EphrinB3) (Fig. 4c).

EphrinB3 behaves as a dependence receptor, which can trigger cell apoptosis [53]. To analyze the potential effect of EphrinB3 on SC survival, we assayed cell viability as above. Incubation of adhered SC with clustered EphrinB3, or Fc as control, for 3 h and 24 h did not induce any morphological difference, and the number of Hoechst^+^pycnotic or caspase3^+^nuclei between Ephrin-treated and control SC remained equivalent (3-h treatment (*n *= 6): Fc: 4.4 ± 2‰, EphrinB3: 4.4 ± 2‰; 24 h post-treatment (*n *= 8): Fc: 9.7 ± 4‰, EphrinB3: 10.4 ± 3‰). Adhesion assays were always performed in serum-free medium, compromising SC proliferation. These results imply that the reduction in the number of attached and spread SC to the different substrates results from their preferential adhesion rather than a toxic effect of the treatment.

To validate the involvement of EphA4 and EphB6 receptors in SC EphrinB3 response, we performed the myelin and EphrinB3 adhesion assay in the presence of antibodies that specifically interfere with EphA4 or/and EphB6 receptors to block SC response. Blocking EphA4 or EphB6 receptor with excess anti-EphA4 or anti-EphB6, respectively, improved SC adhesion and spreading to myelin compared to PBS (Fig. 4d) or to EphrinB3 compared to Fc (Fig. 4e). Ratio of adhered cells myelin/PBS: IgG: 0.4 ± 0.2; anti-EphB6: 0.9 ± 0.4, anti-EphA4: 0.8 ± 0.2. Ratio of adhered cells EphrinB3–Fc: IgG: 0.5 ± 0.09, anti-EphB6: 0.9 ± 0.2, anti-EphA4: 0.6 ± 0.2. Ratio of round cells myelin/PBS: IgG: 1.8 ± 0.9, anti-EphB6: 1.0 ± 0.4, anti-EphA4: 0.8 ± 0.4. Ratio of round cells EphirnB3–Fc: IgG: 1.4 ± 0.3, anti-EphB6: 1.0 ± 0.4, anti-EphA4: 1.2 ± 0.4. Data are expressed as ratio of myelin or EphrinB3 surfaces compared to intra-coverslip control: non-coated surfaces (PBS) or with equimolar concentration of Fc, respectively. However, no additive effect on adhesion was observed when antibodies to both receptors were combined (myelin/PBS: anti-EphA4 + anti-EphB6: 0.8 ± 0.2 (*n* = 11), *p*(IgG vs anti-EphA4 + anti-EphB6) = 0.003; EphrinB3/Fc:anti-EphA4 + anti-EphB6: 0.8 ± 0.3(*n* = 10), *p*(IgG vs anti-EphA4 + anti-EphB6) = 0.008) two-tailed t-student test).

### EphrinB3 increases Schwann cell adhesion and migration on the perivascular ECM component, fibronectin, via integrin ß1

**Fig. 5:**
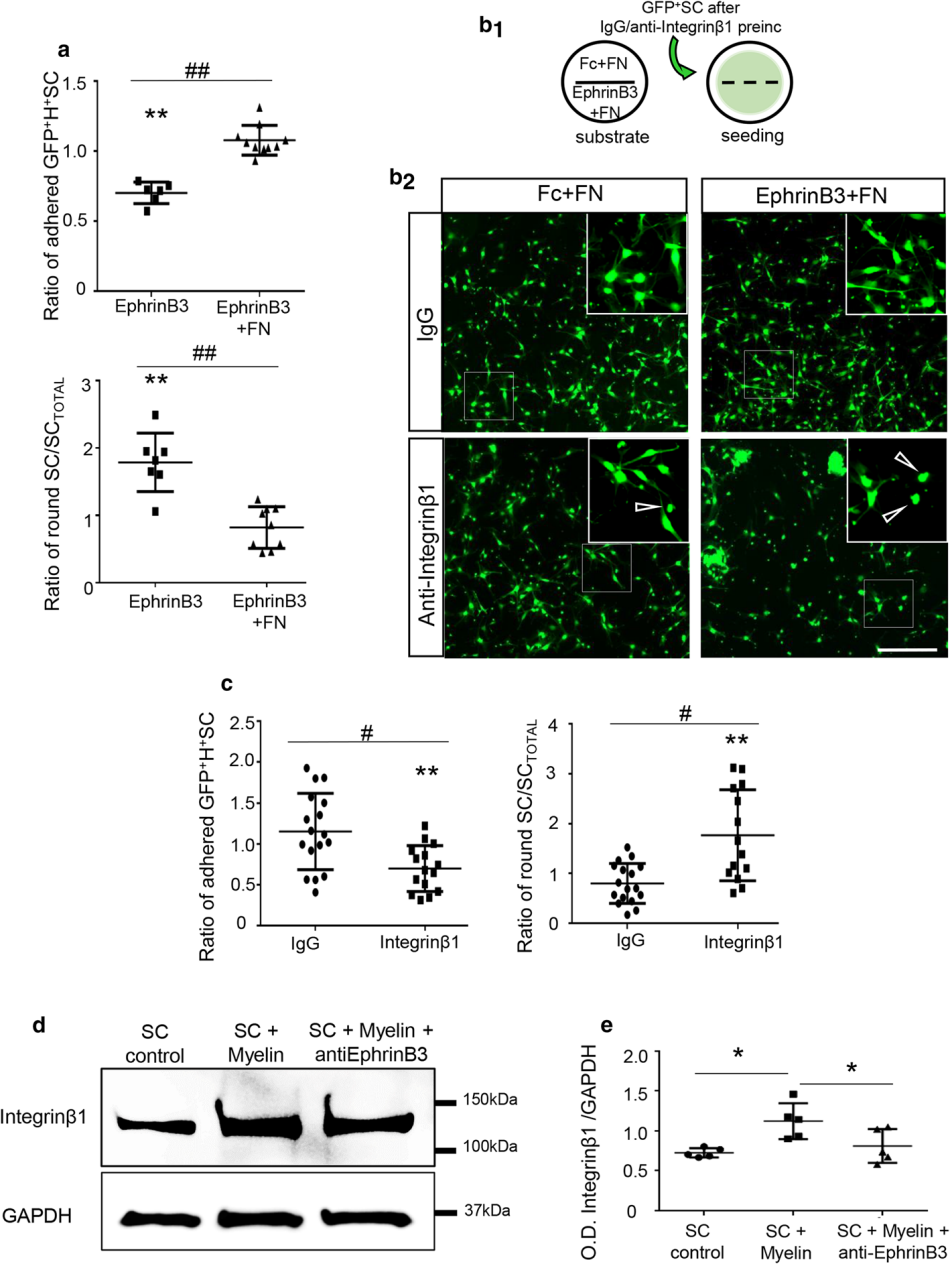
EphrinB3 improves SC adhesion and spreading on FN-coated surfaces. **a** Quantification of the number of adhered and polarized SC seeded on surfaces coated with EphrinB3 with or without FN. Data are expressed as ratio respect to Fc-coated surfaces. EphrinB3 (10 µg/mL), EphrinB3 (10 µg/mL) + FN (2 µg/cm^2^), *n *= 7 per group. **b**_**1**_ Diagram of the adhesion assay on FN. Coverslips divided by silicon strips were coated with Fc + FN (control) or EphrinB3 + FN in each half. After strip removal, cells pre-incubated with IgG or anti-Integrinβ1 were seeded homogenously on the coverslip. **b**_**2**_ Blocking Integrinβ1 decreased SC adhesion and polarization on EphrinB3 + FN compared with Fc + FN. Arrows show examples of “round cells”, scale bar 200 µm. **c** Quantification of SC adhesion and polarization on EphrinB3 when SC are pre-incubated with IgG or anti-integrinß1, *n *= 15–17 per group. **d**–**e** Western blot shows an increased expression of Integrinβ1 when SC are activated by myelin extracts. One-way ANOVA *p *= 0.011, F(2,12) = 6.62, followed by Tukey’s multiple comparisons test *p *< 0.05, *n* = 5 per group. Data are expressed as mean values ± SD. In **a**, c*/** are used for comparison of a group with its hypothetical mean of 1 by one-sample two-tailed *t*-test, and ^#/##^ for comparison between two different groups by two-tailed Mann–Whitney test.*/^#^*p *< 0.05; **/^##^*p *< 0.001

Eph/ephrin can modulate cellular pathways by regulating cell adhesion, either positively or negatively, depending on the cellular context [46]. Thus, a positive regulation of SC adhesion to ECM and FN might have consequences on their migration capacity on this substrate [54, 60]. As our in vivo study indicated that the grafted SC were embedded in perivascular ECM, we studied the effect of EphrinB3 on SC–ECM binding, in particular FN, that favors SC migration [11]. Unlike SC repulsion by EphrinB3-coated surfaces, EphrinB3 improved SC adherence and process expansion on FN-coated surfaces compared to those coated with FN and Fc (Fig. 5a). Ratio of adhered cells: EphrinB3 (10 µg/mL): 0.7 ± 0.07, EphrinB3 (10 µg/mL) + FN (2 µg/cm^2^): 1.07 ± 0.1. Ratio of round cells: EphrinB3 (10 µg/mL): 1.8 ± 0.4, EphrinB3 (10 µg/mL) + FN (2 µg/cm^2^): 0.82 ± 0.3 (data are expressed as ratio compared to Fc–non-FN-coated surfaces).

The main FN-binding receptor on SC is the integrin heterodimer α5β1 [38], and the integrin family is involved in the Eph/ephrin response [20, 60]. To question whether integrinß1 could mediate the mechanism by which EphrinB3 regulates SC–FN binding, we performed an integrinβ1-interfering assay prior to SC adhesion on FN + EphrinB3 (Fig. 5b, c: IgG: 1.1 ± 0.4; anti-integrinβ1: 0.7 ± 0.3. Data are expressed as ratio over control (Fc + FN substrate)). Anti‐integrinβ1 pre-incubation with a specific blocking antibody [34] consistently prevented the increased SC adhesion to FN + EphrinB3-coated surfaces compared to the FN + Fc-coated ones. Moreover, western blot showed that incubation of SC during 30 min with myelin protein extracts (100 µg/mL) increased the expression of Integrinβ1 and this increase was prevented by pre-incubation of the myelin protein extract with anti-EphrinB3 (O.D. of Integrin β1/GAPDH in control: 0.72 ± 0.05, myelin: 1.12 ± 0.22, and myelin + anti-EphrinB3: 0.81 ± 0.21) (Fig. 5d, e). This suggests that myelin-associated EphrinB3 induces integrinβ1 expression, which is involved in the increased ECM adhesion induced by EphrinB3.

**Fig. 6:**
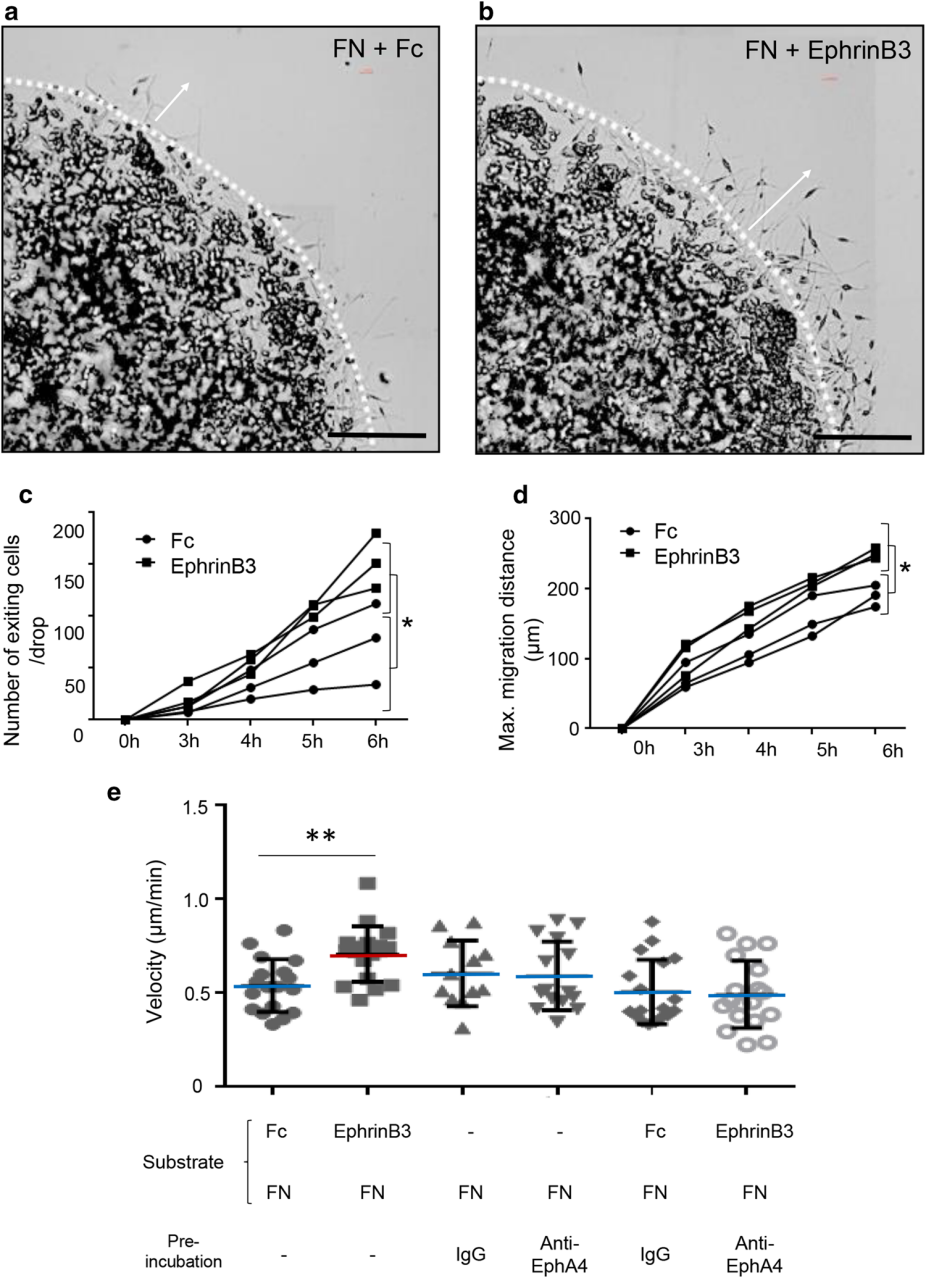
EphrinB3 improves SC migration on FN-coated surfaces. **a**, **b** Exit of SC entrapped in an agarose drop and seeded on FN + Fc (**a**) and FN + EphrinB3 (**b**), scale bar 200 µm. (**c**) More SC exit the agarose drop 5 h post-seeding on FN + EphrinB3 than on control surface (two-way ANOVA with repeated measures: *p* = 0.03, F(1,4) = 10.24). **d** SC migrate over longer distances from the drop-edge 4 h post-seeding (two-way ANOVA with repeated measures: *p* = 0.02, F(1,4) = 12.74) on FN + EphrinB3-coated surfaces (*n* = 3 per group). Graphs represent the values of separate experiments (mean ± SD). **e** Velocity of single SC was measured in different conditions. Only SC seeded on FN + EphrinB3 show a significant increase of migration speed. This increase is reverted when SC are pre-incubated with anti-EphA4 (one-way ANOVA *p* = 0.002, F(5,93) = 4.1; two-tailed Mann–Whitney test *p* = 0.002 between control FN + Fc and FN + EphrinB3 group, *n* = 12–18 per group). In **e**, *n* represents tracked single cells of three different experiments repeated independently. Data are expressed as mean values ± SD. Single cells were tracked from three different independent experiments, **p < 0.01

We examined whether this induced SC adhesion to FN could have some implication in their ability to migrate on FN using the agarose drop assay [2]. SC were seeded on FN in the presence of EphrinB3 or Fc, and their migration when exiting the agarose drop was followed by time-lapse video-microscopy. Significantly more SC migrated out of the drop when sections were coated with FN + EphrinB3 (Fig. 6b, c) compared to control (Fig. 6a, c). Moreover, based on their maximal distance of migration, SC migrated significantly further on FN + EphrinB3 compared to control (Fig. 6d).

Finally, we performed interference experiments to analyze the migratory behavior of SC incubated or not with anti-EphA4 on FN substrate alone or FN + Fc or FN + EphrinB3. Analysis of the migration velocity of single cells traced by video-microscopy on FN confirmed that SC migrate significantly faster on FN + EphrinB3 substrate, compared to Fc + FN control (Fig. 6e). This increased speed was counteracted when SC were pre-incubated with anti-EphA4 compared to control IgG. Moreover, data confirmed that pre-incubation with IgG or anti-EphA4 did not impair SC speed or pattern of migration on FN whether seeded over Fc or EphrinB3 (Fig. 6e).

### Pre-treatment with anti-Eph4 promotes Schwann cell to intermingle more with myelin in vivo and reduces their interaction with blood vessels

**Fig. 7:**
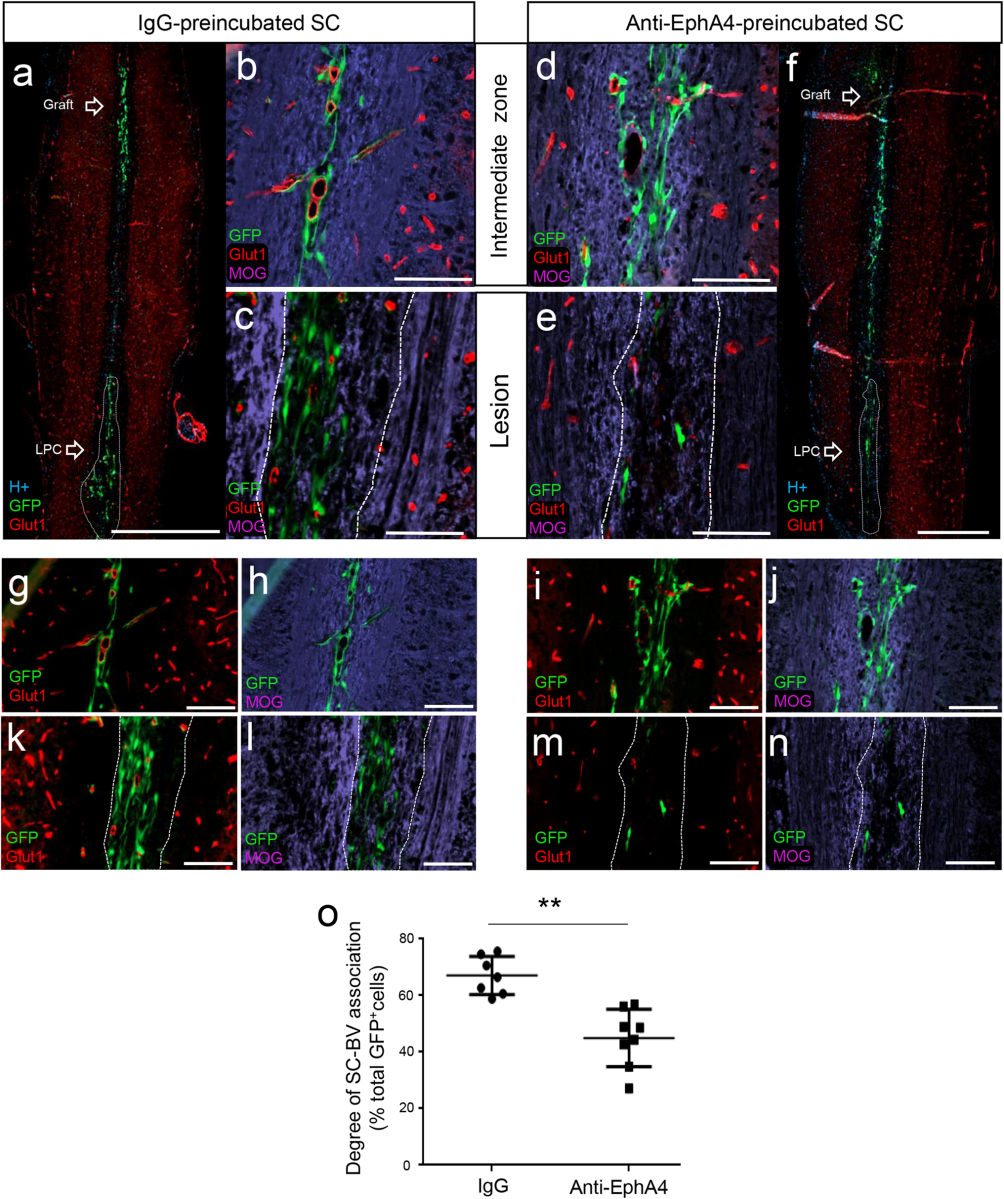
Pre-incubation of SC with anti-EphA4 reduces their migration along BV after transplantation in the demyelinated spinal cord. **a** GFP^+^SC pre-incubated with IgG migrate from the graft along the midline in association with Glut1^+^BV (**b**, **g**) but avoid MOG^+^ myelin (**b**, **h**) before arrival at the lesion at 5 dpi (**c**, **k**, **l**). **f** Anti-EphA4-pre-incubated GFP^+^SC exiting the graft intermingle more with myelin (**d**, **j**), associate less with BV (**d**, **i**) and frequently fail to reach the lesion site at 5 dpi (**e**, **m**, **n**). **o** Quantification of SC in association or not with BV show significant differences between control (IgG) (*n* = 7) and anti-EphA4 pre-incubated SC (*n* = 8) with fewer cells associated with BV after anti-EphA4 pre-incubation (Mann–Whitney test, *p* = 0.0003). Dashed lines delineate lesions. Data are expressed as mean values ± SD. *p < 0.05. Scale bar 100 µm or, in (**a**, **f**), 1000 µm

In vitro experiments established that EphrinB3 had a dual effect, impairing SC interaction with myelin but improving their interaction with FN via increased integrinß1 expression. Moreover, perturbation experiments indicated that anti-EphA4 treatments did not affect SC migratory behavior. To examine whether EphrinB3 plays a role in their integration/migration into CNS white matter and/or their interaction with BV in vivo, we interfered with EphrinB3 by blocking EphA4 and examined SC interactions with BV and myelin. Since no additive effect was observed in vitro when blocking two different receptors, only EphA4 was used. Pre-incubation of SC with the functional anti-EphA4 blocking antibody, prior to transplantation as above, disrupted SC interaction with BV (Fig. 7a–c compared to d–f), evaluated by the number of SC–BV associations (Fig. 7o), and improved their intermingling with myelin along the path of migration (Figs. 7g, h vs 8i, j). While 67% of SC were associated with BV in the control group, only 45% were associated with BV in the anti-EphA4-treated group (Fig. 7o). Moreover, for the same graft–lesion distance, lesion size and amount of grafted cells, more GFP^+^SC were found in the lesion site at 5 dpi in animals grafted with IgG-treated SC (Fig. 7c, k, l) compared to those grafted with anti-EphA4-treated SC (Fig. 7e, m, n, Table 3). Thus, anti-EphA4 treatment reduced the capacity of SC to progress efficiently along vessels and/or enhanced their sensitivity to other myelin inhibitors.

**Fig. 8:**
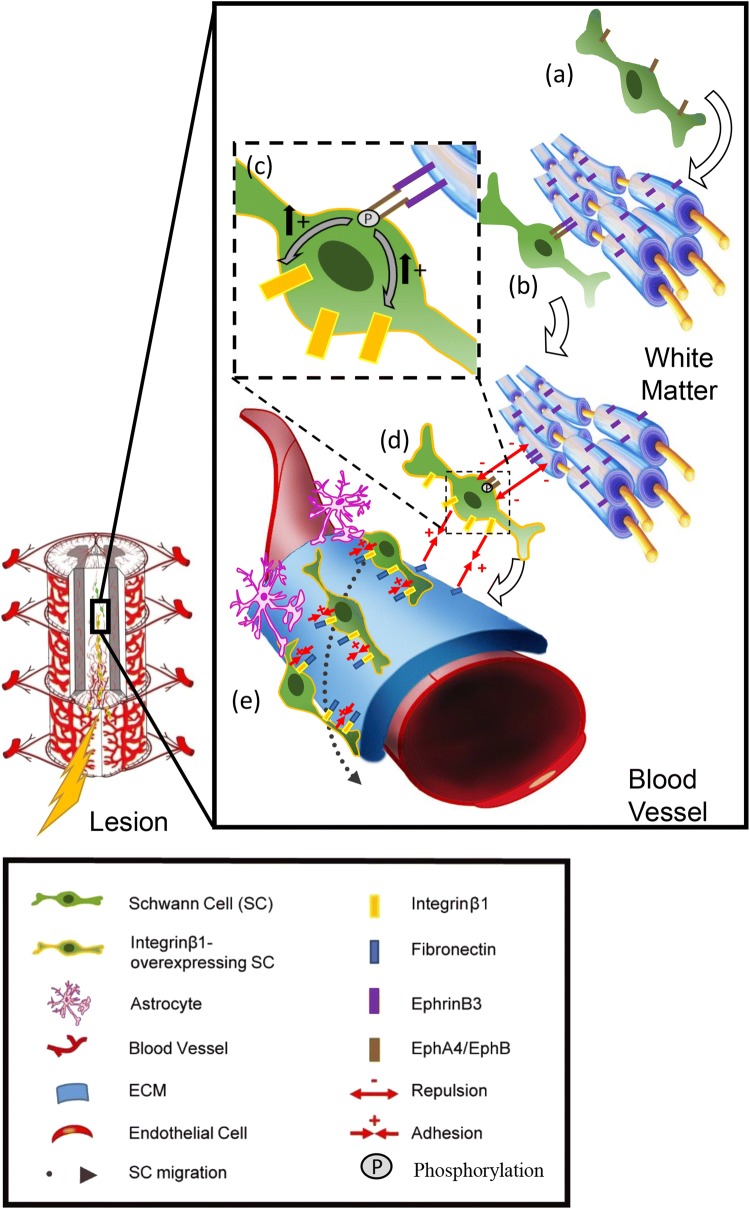
Model of the mechanism of guidance and migration of SC after CNS demyelination. **a**, **b** SC encountering CNS white matter are activated by the myelin-associated EphrinB3 through EphB6- and EphA4-SC receptors. **c**, **d** The activation of these receptors by phosphorylation impairs SC adhesion to white matter and increases SC expression of Integrinβ1, promoting their adhesion to BV extracellular matrix. **e** Lesions of white matter undergo the formation and/or remodeling of BV which increases expression of ECM adhesion molecules, such as FN, and further facilitate SC mobilization towards the lesion

**Table 3:** Extent of migration of grafted GFP^+^SC after interference of Eph signaling

	Distance graft–LPC injections (mm)	Lesion size (mm^2^)	SC distance cells towards lesion (mm)	Animals with SC in the lesion (%)
lgG (control)	4.14 ± 0.69	4.00 ± 1.73	4.57 ± 0.79	100
Anti-EphA4	4.50 ± 0.53	3.25 ± 1.91	2.53 ± 1.95*	37.5

The reduced extent of migration by SC pre-incubated with anti-EphA4 prior to grafting compared to control SC (Mann–Whitney test, *p* = 0.044) correlates with a reduced percentage of animals in which grafted SC are recruited by the lesion. Data are expressed as mean values ± SD. *p < 0.05

## Discussion

Compelling evidence indicates that SC, whether recruited from the periphery or from resident progenitor cells, remyelinate CNS axons after demyelination [6, 43, 59, 61, 62], having an obvious impact on clinical recovery [24, 26]. However, in spite of SC presence within the CNS, the modalities of their recruitment after myelin injury remain elusive. To gain insights into this question, we studied SC behavior when confronted with CNS myelin and BV ex vivo and in vivo in demyelinating conditions. Using classic, live, and 3D imaging as well as electron microscopy, we provide solid evidences that SC use the vascular scaffold to migrate within the adult demyelinated CNS. This phenomenon is doubly modulated by CNS myelin, and in particular, the CNS myelin-specific component, EphrinB3, which negatively regulates SC adhesion to, and spreading on, myelin, while enhancing SC adhesion to perivascular ECM.

Communication and coordinated interaction between the vascular and nervous systems [29, 50] results in a functional neurovascular unit that contributes to wound healing [16], immune response [21] and embryonic development [40]. Recently, a new role in supporting long-distance migration of different kinds of cells within the nervous system was attributed to BV, both during development and adulthood [15, 58] as well as under pathological conditions [16, 28]. So far, despite the increasing number of cells guided by BV, the role of these structures in guiding SC within the CNS to participate in CNS repair has not been explored. While SC are known to promote endothelial cell migration and angiogenesis [31] and use BV as scaffold during peripheral nerve regeneration [16], our work demonstrates that this SC–BV interface extends to the CNS and is of relevance to their contribution to CNS repair.

We show that BV are a favorable substrate for exogenous SC and guide them towards demyelinating lesions. BV serve as scaffold for SC as soon as they leave the graft and along their path until arrival at the lesion. Along this path, SC are organized in chains going from one BV to another. At their arrival in the lesion, SC embedded in perivascular ECM become more randomly dispersed and this dispersal faithfully overlaps with BV expansion (Fig. 1c_2_). While associated with BV at their arrival at the lesion at 3 dpi, SC detached from BV to contact and align with the demyelinated axons at 5 dpi. This change in substrate association may result from signals arising from the axons that trigger their differentiation into more mature SC as a first step to myelin repair. The absence of SC away from their narrow path of migration between the graft and lesion, as well as their progressive increase in number in the lesion, point to their specific recruitment by the lesion most likely involving attractant signals yet to be defined.

SC intervening in CNS repair are generated mainly from endogenous OPCs nested within perivascular niches while only a small proportion originate from PNS sources [6, 61]. Our model of grafting SC remotely (4 mm) from the lesion shows that once they are in the CNS, SC can travel long distances on BV to reach the lesion. Based on the cited work, long-distance migration along BV may then reflect the behavior of the majority of SC participating to endogenous remyelination. Moreover, the induction of demyelination in *Krox20*^*Cre*^*,Rosa*^*YFP*^ mice showed that YFP^+^ SC were associated with BV in vicinity of the lesion, therefore, implying that YFP^+^ SC, irrespective of their PNS or CNS origin, use BV as scaffold to reach the lesion during spontaneous repair. Due to technical limitations and the difficulty to track a minor event, our work did not reveal the modalities of SC transgression between the PNS and CNS. However, the observation of SC migration over long distances on BV when ectopically placed in the CNS, and the presence of a vascular network forming bridges between the PNS and CNS, hints towards the possibility that PNS-derived SC can use this scaffold to transgress the PNS–CNS border. Future studies based on more specific transgenic tools and other lesion models are needed to resolve this question.

BV also support SC migration in the injured PNS. However, in these circumstances, SC create direct contacts via protrusion with endothelial cells to migrate from one nerve stump to the other or in vitro when grown in 3D, suggesting that these protrusions constitute mechanical means to propel SC migration along BV in a tight environment [16]. Although we found SC associated with BV along their migrating route, direct contact with endothelial cells or pericytes was never observed. Instead, SC were heavily embedded in the perivascular ECM, thus indicating that although SC share similar mechanisms to conquer the injured nervous system, some differences exist in their mode of migration between CNS and PNS, and BV guidance and perivascular ECM seems to prevail for their migration in the CNS. The observed differences may result from different molecular and cellular environment existing between PNS and CNS, including different degrees of confinement in which SC are placed.

We previously demonstrated that SC avoid and are repelled by myelin [10, 17]. Here, we confirm these data and show that SC, prevented from migrating directly through white matter, are somehow forced to migrate on BV. We showed previously that MAG, a CNS myelin component that prevents axonal regeneration in the CNS, is also inhibitory to SC migration and survival [17]. While MAG accounted only partially for the repulsive effect of myelin to SC, we identified EphrinB3 as another myelin component negatively regulating SC in contact with CNS myelin. Like MAG, EphrinB3/EphA4 receptor signaling has been implicated in axon pathfinding [49]. This suggests that myelin components exerting their inhibitory effect on axons are not exclusively directed against axons, but extend their inhibition to other neural components such as myelin-competent cells, preventing differentiation of oligodendrocyte progenitors into mature oligodendrocytes [36] as well as SC survival and migration (present data).

We first proved in vitro that SC-bound EphrinB3 is able to activate both EphA4 and EphB1 by phosphorylation, as well as to bind EphB6, which can be also trans-phosphorylated EphB1 [27]. Once bound to these receptors, EphrinB3 impairs the adhesion of these cells to myelin proteins, diminishing their process extension onto their substrate. Our in vitro data indicate that interfering with both EphB6 and EphA4 does not show additional improvement of this myelin–SC repulsion compared to single interference. Eph receptor signaling is not as straightforward as one receptor binding to one ligand. To trigger efficient activation, Eph receptors must cluster, homotypically (one subtype of Eph receptor) or heterotypically (involving the oligomerization of different Eph receptor subtypes) [56]. This lack of additive effect suggests that the mechanism of Eph/ephrin activation in SC might be mediated by heterotypic recruitment independent of the initial receptor activation.

Ephrin signaling not only induces repulsion but also modulates expression of adhesion molecules [5, 20, 51]. We show that myelin-associated EphrinB3 modulates SC adhesion and migration to ECM, in particular FN, and enhanced integrinβ1 expression, overruling SC inhibition by myelin in vitro and promoting their migration along BV to reach the demyelinated lesions in vivo. This event occurs in correlation with increased FN expression, among other ECM molecules, during BV remodeling in response to demyelination (data not shown Garcia-Diaz, unpublished data) [59], suggesting that the increased expression of ECM molecules by BV favors SC–BV interaction and subsequent migration along the vasculature in vivo.

Despite the present implication of EphA4 and EphB6 receptors in SC response to myelin, and their contribution to SC migration along CNS BV, the involvement of other Eph receptors in SC migration within the CNS should not be disregarded. Of note, EphB2, also expressed by SC and able to bind myelin-associated EphrinB3, mediates SC–SC interaction through N-cadherin re-localization to organize SC chain migration in the PNS [51]. This may be implicated in the SC chains forming bridges between BV (Fig. 1c_1_ and f, Movie S1).

In addition, EphA4 is involved in SC–astrocyte repulsion [1]. Although the interactions between SC and astrocytes have not been explored in this study, the localization of the grafted SC in the vascular unit between the blood vessel wall and the astrocyte feet suggests that the repulsion exerted by astrocytes can help confine SC to the perivascular space, and thereby contribute to the mechanism of SC migration along BV. Interfering with EphA4 in SC could have altered this interaction and further allowed the grafted cells to escape from their perivascular path and intermingle more with the surrounding white matter parenchyma.

In conclusion, we used multiple in vivo and in vitro approaches to highlight a novel mechanism of guidance and migration of SC during the early events of CNS repair. We also provide strong evidences that the Eph/ephrin family regulates the complex interactions existing between SC, myelin and blood vessels. SC encountering myelin-associated EphrinB3 retract their processes failing to intermingle with white matter, and adhere preferentially to BV via activation of Integrinβ1. This dual effect, repulsing SC from CNS myelin and enhancing their attraction to basal lamina, directs their migration along CNS vasculature towards the lesion (Fig. 8). Lesions of white matter undergoing the formation and/or reshaping of the vasculature with increased expression of ECM adhesion molecules, in particular FN, further triggers SC mobilization throughout the lesion. While SC invasion of the CNS is not restricted to demyelinating diseases, future studies should indicate whether such mechanisms are of relevance for other clinical pathologies such as trauma as well as genetic and acquired myelinopathies.

## Electronic supplementary material

Below is the link to the electronic supplementary material.

Suppl.Fig. 1. Perivascular migration of endogenous SC in response to demyelination. (a,b) MBP, Glut1 and YFP immunostainings of control *Krox20*^*Cre/*+^*R26R*^*YFP/*+^spinal cord sections show the absence of YFP^+^SC in spinal cord without lesion. (c,d) General view of YFP^+^SC on Glut1^+^ BV near the lesion at 3 dpi. (e,f) Examples of YFP^+^ SC associated with Glut1^+^ BV and expressing Sox10. Insets show separate colors for YFP and Sox10. e is an enlargement of the boxed area in d. (g) YFP^+^ cells do not co-label with the oligodendroglial marker Olig2. (h,i) YFP^+^ cells (green) sometimes express (red arrows) the microglial marker Iba1 (white). Scale bar in a–d: 100 µm, in e–f: 20 µm. Suppl.Fig. 2. Glut1 specifically labels endothelial cells. (a–c) Co-labeling of Glut1 with the endothelial marker CD31 (d–l). Absence of co-labeling of Glut1 and the microglial/macrophages markers CD68, CD11b and F8/40(d–f), the SC marker p75(g-i), and the pericyte marker CD13 (j–l). Scale bar 10 µm. Suppl.Fig. 3. Time-lapse imaging of SC movements on blood vessels. (a) Grafted GFP^+^SC (green) in the spinal cord reach rhodamine–lectin-labeled BV (red). The white arrowhead follows a GFP^+^SC gliding along a large BV. The blue arrowhead follows a GFP + SC jumping from one BV branch to another (movie S1). (b) GFP^+^SC tend to migrate in chain on BV, but some (white arrowhead) escape and move on the outer BV surface. The asterisk identifies a SC that is less motile (movie S2). Scale bar 100 µm. (PDF 752 kb)

Supplementary material 2 (AVI 1231 kb)

Supplementary material 3 (AVI 1503 kb)
