# Supplementary figures and images for: Blood vessels guide Schwann cell migration in the adult demyelinated CNS through Eph/ephrin signaling
Source: Acta Neuropathol. 2019 Apr 22;138(3):457–76. doi: 10.1007/s00401-019-02011-1 (PMC6689289; doi:10.1007/s00401-019-02011-1)

Suppl. Fig. 1

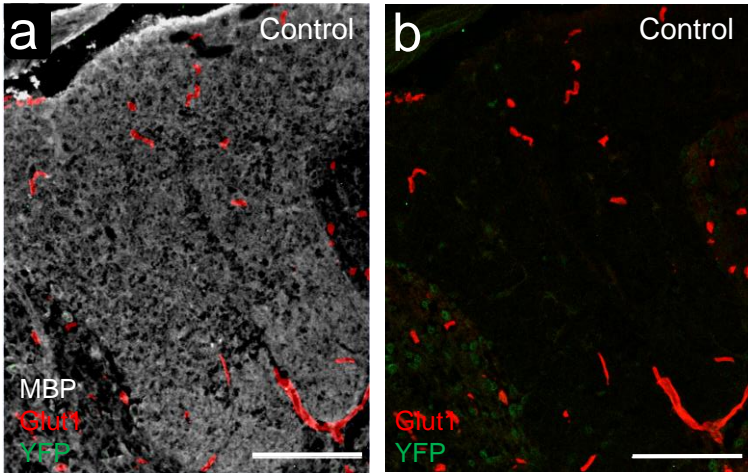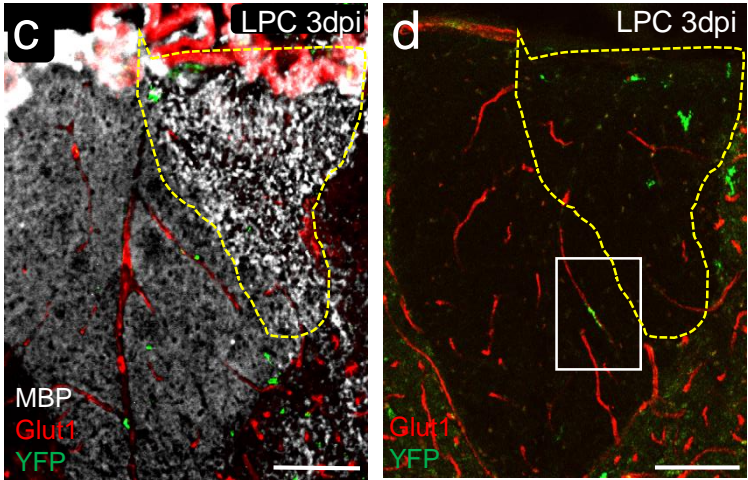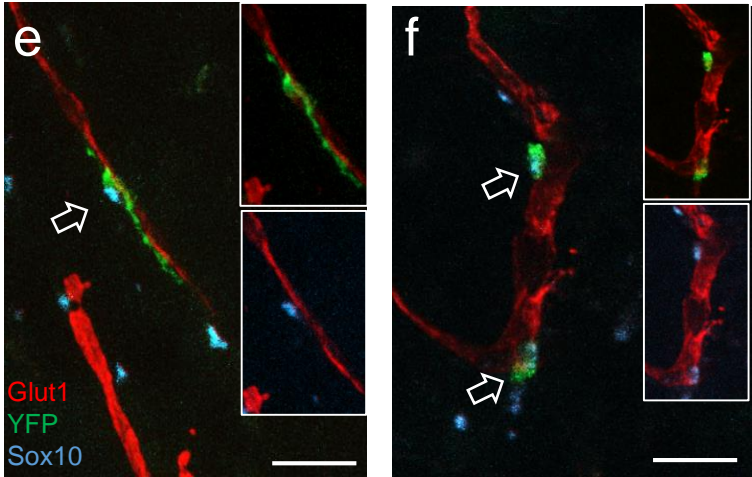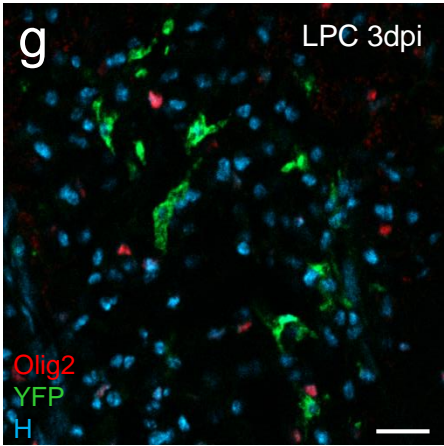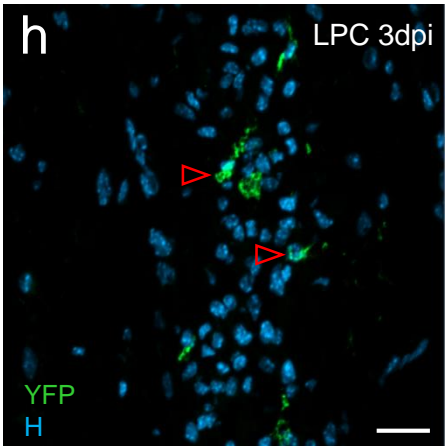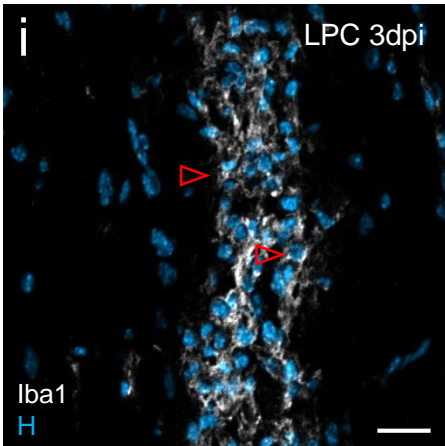

Suppl. Fig. 2

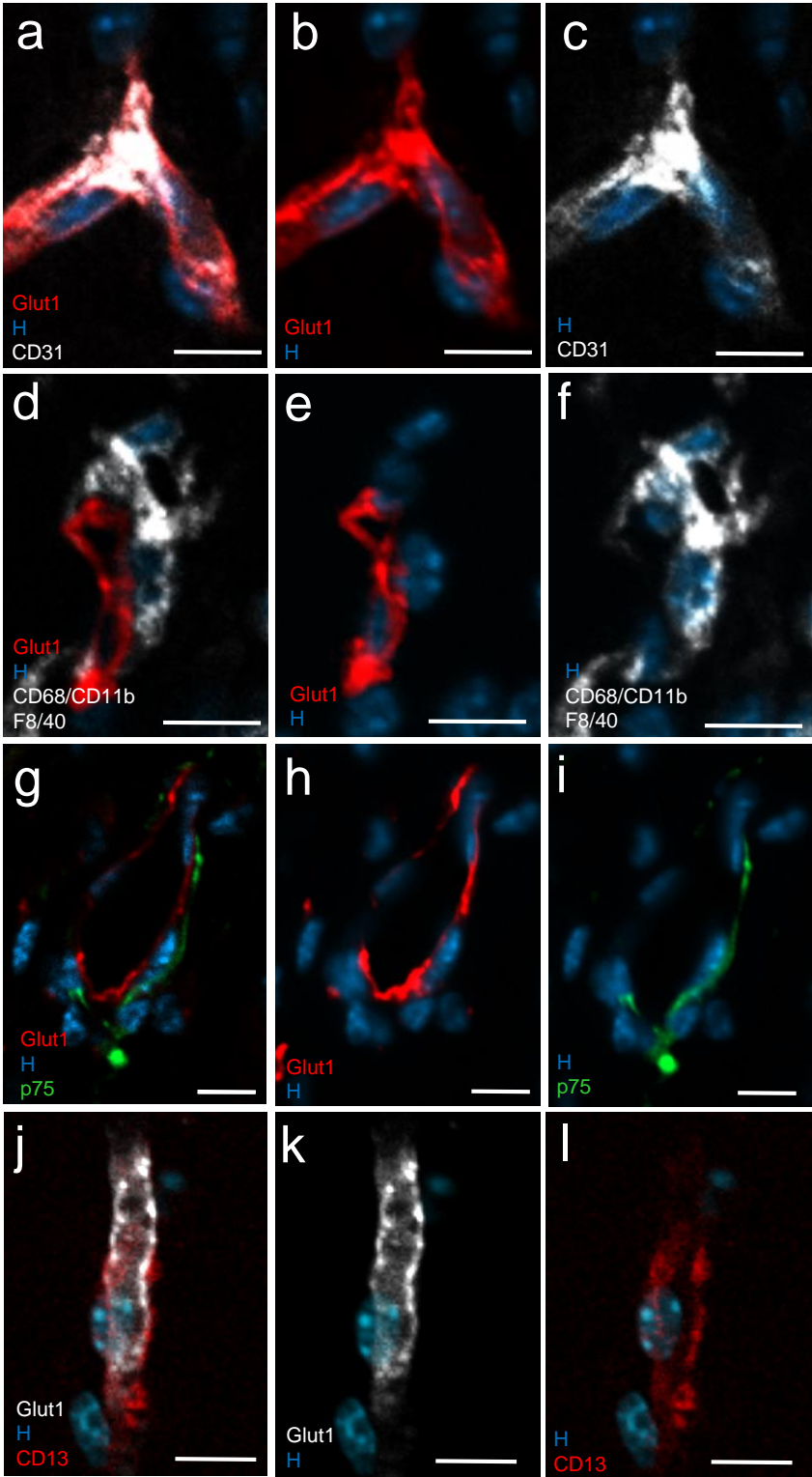

Suppl. Fig. 3

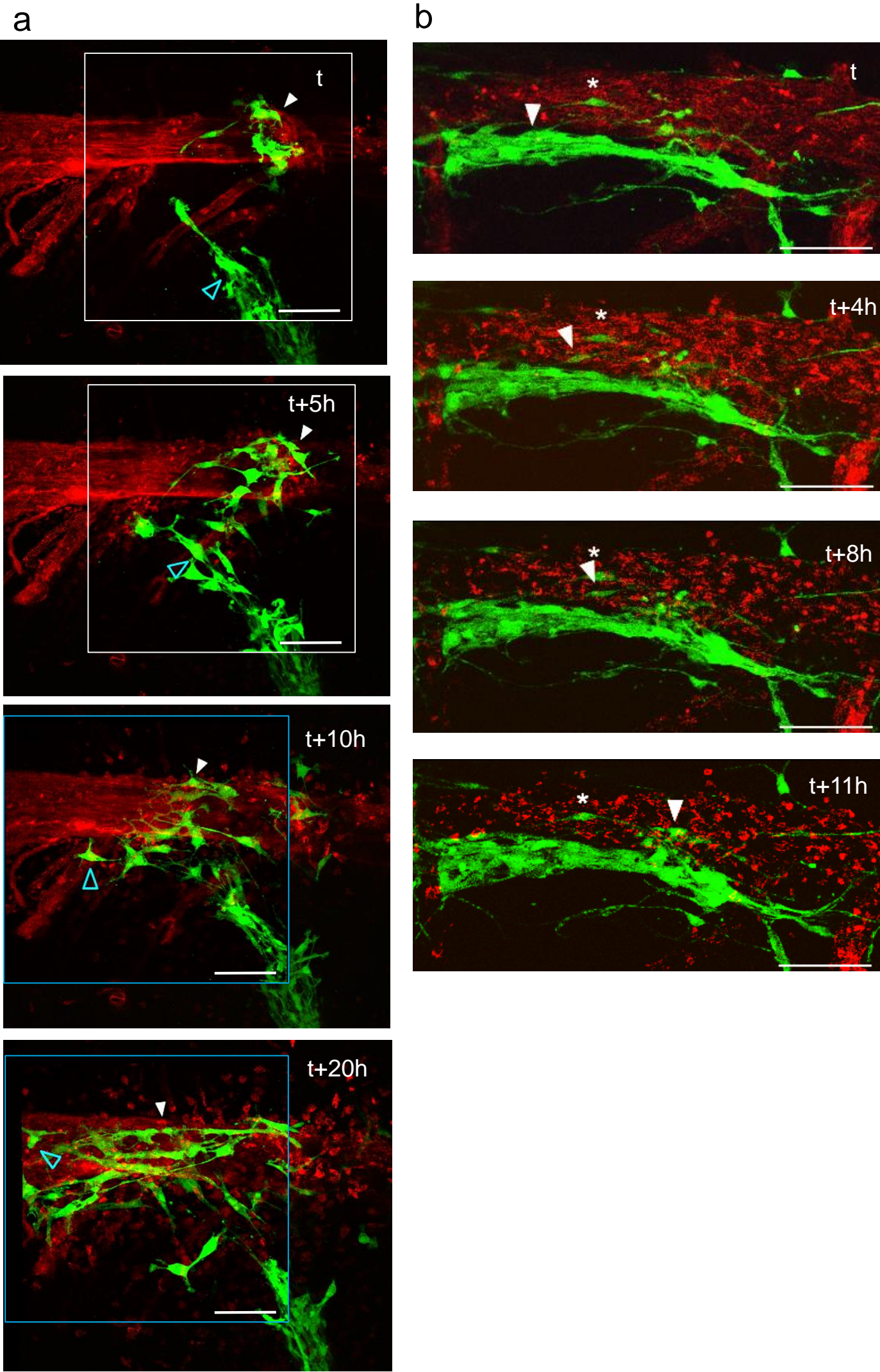

Supplement: Supplementary file 1 — Suppl.Fig. 1. Perivascular migration of endogenous SC in response to demyelination. (a,b) MBP, Glut1 and YFP immunostainings of control Krox20Cre/+R26RYFP/+spinal cord sections show the absence of YFP+SC in spinal cord without lesion. (c,d) General view of YFP+SC on Glut1+ BV near the lesion at 3 dpi. (e,f) Examples of YFP+ SC associated with Glut1+ BV and expressing Sox10. Insets show separate colors for YFP and Sox10. e is an enlargement of the boxed area in d. (g) YFP+ cells do not co-label with the oligodendroglial marker Olig2. (h,i) YFP+ cells (green) sometimes express (red arrows) the microglial marker Iba1 (white). Scale bar in a–d: 100 µm, in e–f: 20 µm. Suppl.Fig. 2. Glut1 specifically labels endothelial cells. (a–c) Co-labeling of Glut1 with the endothelial marker CD31 (d–l). Absence of co-labeling of Glut1 and the microglial/macrophages markers CD68, CD11b and F8/40(d–f), the SC marker p75(g-i), and the pericyte marker CD13 (j–l). Scale bar 10 µm. Suppl.Fig. 3. Time-lapse imaging of SC movements on blood vessels. (a) Grafted GFP+SC (green) in the spinal cord reach rhodamine–lectin-labeled BV (red). The white arrowhead follows a GFP+SC gliding along a large BV. The blue arrowhead follows a GFP + SC jumping from one BV branch to another (movie S1). (b) GFP+SC tend to migrate in chain on BV, but some (white arrowhead) escape and move on the outer BV surface. The asterisk identifies a SC that is less motile (movie S2). Scale bar 100 µm. (PDF 752 kb) [file 401_2019_2011_MOESM1_ESM.pdf]
